# Discovery of a Potent Dual Inhibitor of Aromatase and Aldosterone Synthase

**DOI:** 10.1021/acsptsci.3c00183

**Published:** 2023-11-23

**Authors:** Annachiara Tinivella, Marta Banchi, Guido Gambacorta, Federica Borghi, Paola Orlandi, Ian R. Baxendale, Antonello Di Paolo, Guido Bocci, Luca Pinzi, Giulio Rastelli

**Affiliations:** † Department of Life Sciences, University of Modena and Reggio Emilia, Via G. Campi, Modena 41125, Italy; ‡ Department of Clinical and Experimental Medicine, University of Pisa, Via Roma 55, Pisa 56126, Italy; § Department of Chemistry, University of Durham, Lower Mount Joy, South Rd, Durham DH1 3LE, U.K.

**Keywords:** drug design, aromatase inhibitors, aldosterone synthase inhibitors, breast cancer, cardiovascular disease, multi-target Inhibitors, benzylimidazole derivatives

## Abstract

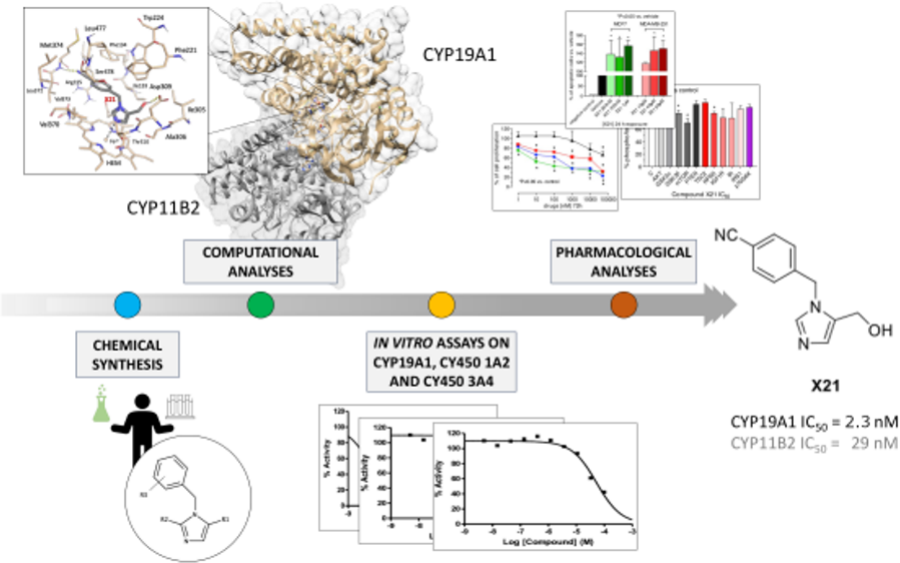

Estrogen deficiency derived from inhibition of estrogen biosynthesis is a typical condition of postmenopausal women and breast cancer (BCs) patients undergoing antihormone therapy. The ensuing increase in aldosterone levels is considered to be the major cause for cardiovascular diseases (CVDs) affecting these patients. Since estrogen biosynthesis is regulated by aromatase (CYP19A1), and aldosterone biosynthesis is modulated by aldosterone synthase (CYP11B2), a dual inhibitor would allow the treatment of BC while reducing the cardiovascular risks typical of these patients. Moreover, this strategy would help overcome some of the disadvantages often observed in single-target or combination therapies. Following an in-depth analysis of a library of synthesized benzylimidazole derivatives, compound **X21** was found to be a potent and selective dual inhibitor of aromatase and aldosterone synthase, with IC_50_ values of 2.3 and 29 nM, respectively. Remarkably, the compound showed high selectivity with respect to 11β-hydroxylase (CYP11B1), as well as CYP3A4 and CYP1A2. When tested in cells, **X21** showed potent antiproliferative activity against BC cell lines, particularly against the ER+ MCF-7 cells (IC_50_ of 0.26 ± 0.03 μM at 72 h), and a remarkable pro-apoptotic effect. In addition, the compound significantly inhibited mTOR phosphorylation at its IC_50_ concentration, thereby negatively modulating the PI3K/Akt/mTOR axis, which represents an escape for the dependency from ER signaling in BC cells. The compound was further investigated for cytotoxicity on normal cells and potential cardiotoxicity against *h*ERG and Nav1.5 ion channels, demonstrating a safe biological profile. Overall, these assays demonstrated that the compound is potent and safe, thus constituting an excellent candidate for further evaluation.

Nowadays, breast cancer (BC) is the most-commonly diagnosed malignant cancer in women, accounting for 36% of oncological patients.^1,2^ The incidence of BC is steadily increasing. Despite the progress in early diagnosis and treatment, which have improved survival rates, continued research into new therapies is still needed.^2^ BC is classified as hormone receptor positive,^3,4^ based on the expression of estrogen receptor (ER), progesterone receptor (PR), and human epidermal growth factor receptor-2 positive (ERBB2/HER2+).^5^ Approximately 70% of diagnosed BCs are ER+, and prolonged exposure to hormones is known to induce cancer.^4^ ER+ patients are clinically treated with (i) selective estrogen receptor modulators (SERMs), such as tamoxifen; (ii) selective estrogen receptors degraders (SERDs); or (iii) aromatase inhibitors (AIs). AIs inhibit a key enzyme for the conversion of androgens to estrogens.^6,7^ The 4-hydroxy metabolite of tamoxifen competitively binds to ER in BC cells, thereby inhibiting transcription and ensuing mitogenic effects in both pre- and postmenopausal women.^8,9^ AIs suppress aromatase activity, thus, decreasing circulating estrogen levels and preventing BC cells from proliferation. AIs are usually employed as a second line of treatment in tamoxifen-resistant tumors and are effective only in postmenopausal women, who represent the majority of BC patients.^4^ The discouraging risk/benefit profile of tamoxifen has prevented the use of this drug for periods longer than 5 years, and severe toxicities, including endometrial cancer and thrombosis, have been observed.^9^ In contrast, AIs have shown better efficacy and tolerability in comparison with tamoxifen, thus becoming the first choice as adjuvant therapy for postmenopausal women.^8^ Unfortunately, most patients who survive cancer die from other compromised health conditions, in particular cardiovascular diseases (CVDs).^10^ Indeed, estrogens plays an important role in protecting the heart, preventing heart failure, post myocardial infarction and ventricular hypertrophy and remodeling,^11−14^ while also preventing kidney issues.^15^ The low estrogen levels typical of menopausal women are further reduced by treatment with AIs in BC patients, leading to major risks of CVDs. Long-term estrogen deficiency after AIs treatment also influences the physiological functions of estrogens and leads to changes in lipid profiles as well as bone loss.^16^ Altered lipid profiles are a possible contributor to the increased risk of CVD in these patients, although this can be partially managed with antihyperlipidemic drugs.^17^ In addition, low levels of estrogens negatively interfere with the Renin–Angiotensin–Aldosterone system (RAAS) by increasing the concentration of all components of the RAAS, especially aldosterone, because high levels of renin, angiotensin II (Ang II), angiotensin-converting enzyme (ACE), and angiotensin type 1 receptor (AT1R) further stimulate aldosterone biosynthesis.^18−23^ Aldosterone excess (hyperaldosteronism) leads to kidney, brain, blood vessel, and heart complications,^9^ pointing to the need for maintaining balanced plasma aldosterone levels during estrogen deficiency. This effect can be achieved by inhibiting the aldosterone synthase enzyme (CYP11B2), which plays a key role in the biosynthesis of aldosterone by converting 11-deoxycorticosterone to aldosterone.^8,24^

Based on this rationale, this work aims at identifying dual inhibitors of the aromatase CYP19A1 and aldosterone synthase CYP11B2 enzymes as a straightforward way to provide an effective and safer cancer treatment while potentially reducing cardiovascular issues. Indeed, a polypharmacological approach may be more effective and can have several potential advantages over single-target or multiple-drug regimens.^25^ To this end, a library of benzylimidazole compounds was synthesized and analyzed in silico by means of an integrated approach, which included: (i) focused polypharmacology searches made through the LigAdvisor web platform developed in our group;^26^ (ii) 3D ligand-based similarity analyses; and (iii) docking calculations into selected conformations of the CYP19A1, CYP11B2 and CYP11B1 enzymes. The biological evaluation of the best candidates led to the identification of promising compound **X21**, which showed potent and balanced aromatase and aldosterone synthase dual inhibitory activity, high selectivity, promising cellular activity, and no cardiotoxicity, thus constituting an excellent candidate for further evaluation.

## Results and Discussion

### Chemistry

**Figure 1:**
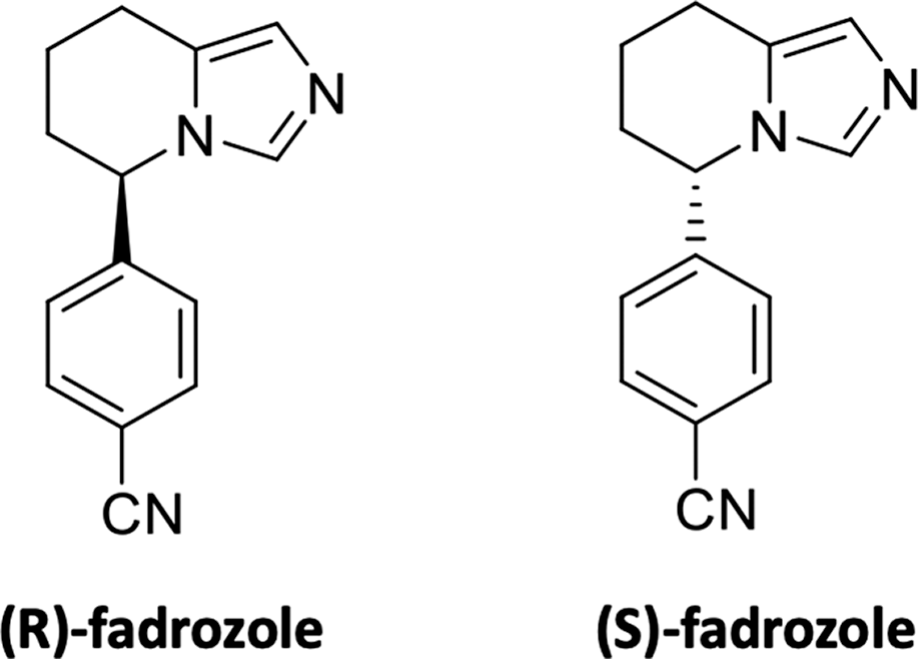
Structures of (*R*)-fadrozole and (*S*)-fadrozole.

**Figure 2:**
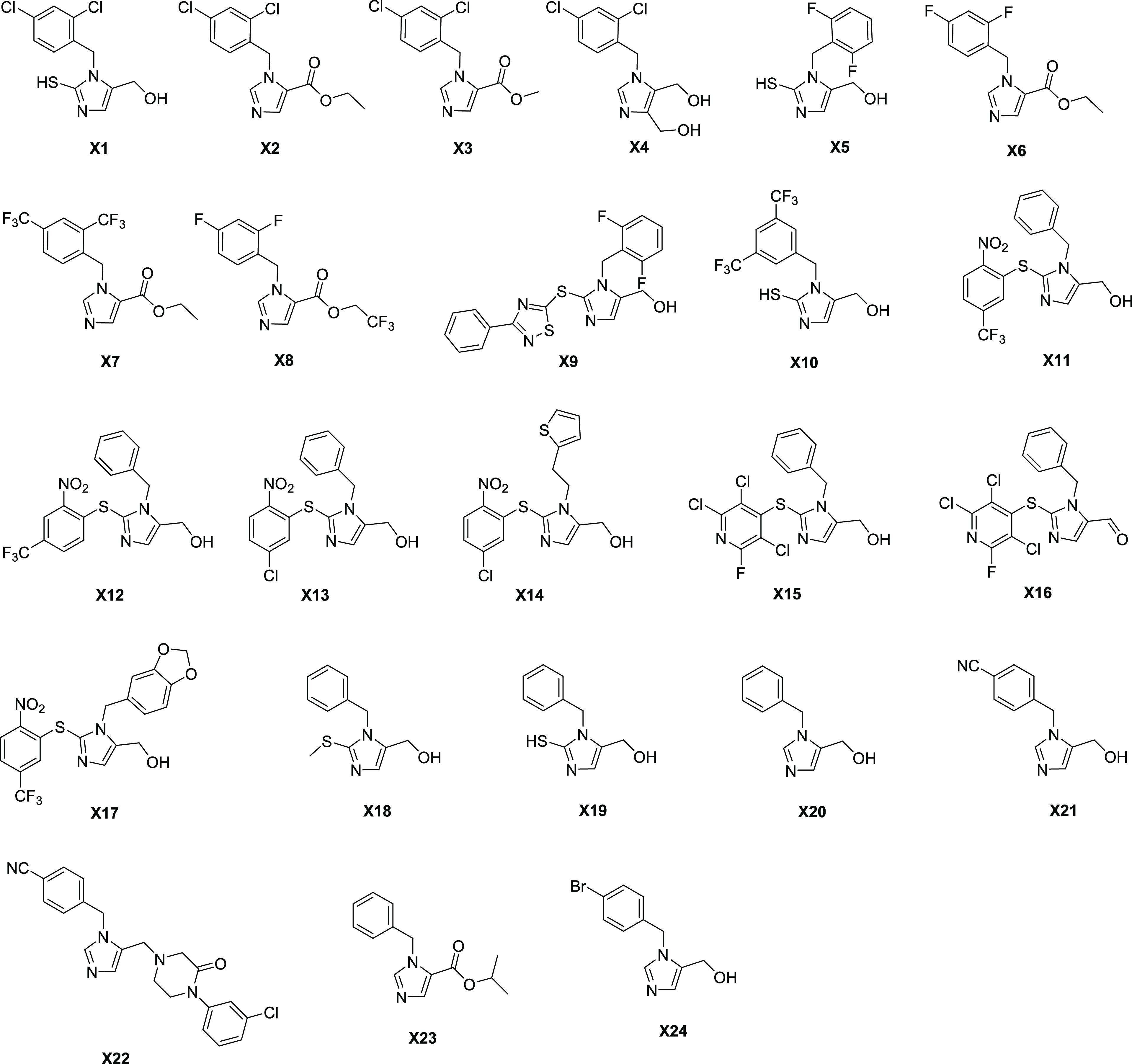
Synthesized library of benzylimidazole derivatives.

Based on the chemical structure and mechanism of action of fadrozole (Figure 1),^27−31^ which is a known CYP19A1 and CYP11B2 inhibitor that however lacks selectivity against CYP11B1, a library of benzylimidazole derivatives (Figure 2) was synthesized and thoroughly investigated.

Figure 1 shows the enantiomers of fadrozole, which display different inhibitory activity. In particular, while (*S*)*-*fadrozole potently inhibits CYP19A1 and CYP11B1 and has lower activity on CYP11B2, (*R*)*-*fadrozole is scarcely active on CYP19A1 and potently inhibits both CYP11B2 and CYP11B1 with no selectivity.^32−34^ This is an important aspect to emphasize in order to better evaluate the potential inhibitory activity of the benzylimidazole derivatives synthesized in this work (Figure 2). In fact, compounds showing unselective inhibition of CYP11B2 and CYP11B1 might provide severe side effects, progressing to acute adrenal insufficiency and potentially fatal cardiovascular collapse.^35^ The compounds were synthesized using previously reported synthetic routes.^36−39^ The functionalized thioimidazole species were assembled through a multicomponent one-pot process based upon a Marckwald reaction.^36^ For example, compound **X1** was synthesized starting from dihydroxyacetone dimer, potassium thiocyanate, and the appropriately functionalized 2,4-dichlorobenzylic amine hydrochloride salt. These thioimidazole derivatives (i.e., **X1**, **X5**, **X10**, and **X19**) were also derivatized, e.g., through: (i) alkylation/S_N_Ar arylation of the nucleophilic thiol^39^ (i.e., **X9**, **X11–X18**); (ii) thiol oxidative cleavage^37^ (i.e., by desulfurization to yield an imidazole core to obtain **X20**, **X21**, and **X24**), and/or Corey–Gilman–Ganem oxidation of the primary alcohol side chain to obtain aldehydes or esters compounds **X2**, **X3**, **X6**–**X8**, and **X23**), or;^37^ (iii) through activation and nucleophilic substitution as in the case of **X22**. Overall, the synthesized library consisted of 24 benzylimidazole derivatives (MW from 190 to 450 Da) variously decorated on the benzene and imidazole rings (Figure 2). Most of the compounds presented at least one substituent on the benzene ring, especially halogens. In these cases, one or two halogens are introduced at each position of the aromatic core. Regarding compounds **X21** and **X22**, a nitrile group was located in *para* position to the methylene bridge, while derivatives **X14** and **X17** presented a thiophene and a 1,3-benzodioxolane ring, respectively. Substitutions on imidazole mainly involved positions 2- and 5-positions. The 2-position was substituted with a reactive thiol group (**X1**, **X5**, **X10**, **X19**) or a thioether, incorporating simple hydrocarbons (**X18**), or substituted aromatic rings (**X9**, **X11**–**X17**). The 5-position was functionalized with primary alcohols or esters. Exceptions were compounds **X16** and **X22**, which presented an aldehyde and a substituted piperazine, respectively. Compound **X4** was the only one of the series to possess an additional primary alcohol at position 4 of the imidazole. In conclusion, the library included molecules having a common substructure but a relatively wide diversity and MW range, owing to the various functional groups present on the benzylimidazole core. This library was analyzed through ligand-based and structure-based computational tools in order to identify the more promising candidates for dual inhibition.

### Computational Analyses

In order to identify the best potential dual inhibitors of CYP19A1 and CYP11B2, the synthesized compounds were investigated in silico with LigAdvisor (https://ligadvisor.unimore.it/, accessed on July second, 2021)^26^ a Web server developed in our group that facilitates polypharmacology and drug repurposing predictions.^25,40,41^ In particular, LigAdvisor implements ECFP4 (circular–equivalent to Morgan) and MACCS fingerprints-based searches on DrugBank^42^ and Protein Data Bank (PDB)^43^ ligands, which are less populated by pan-assay interference and potential false-positive compounds. The performed 2D-similarity analyses highlighted a significant degree of similarity between compounds **X21** and **X12** with anastrozole (DB01217) and levoketoconazole (DB05667) (Table S1). Of note, anastrozole is a potent AI, while levoketoconazole shows significant activity against aromatase, CYP11B2 and CYP11B1.^44−48^ Compounds **X2**, **X3**, **X6**, and **X8** resulted to be similar to the highest number of DrugBank compounds with reported activity annotations on CYP19A1, CYP11B2, and CYP11B1 (Tables 1 and S1), whereas compounds **X21** and **X22** resulted to be similar to the highest number of PDB ligands according to ECFP4*fp* fingerprints (Tables 1 and S1). In particular, **X21** resulted significantly similar to (S)*-*fadrozole (PDB ligand ID: JD7),^49^ osilodrostat (PDB ligand ID: YSY),^50^ and (R)-fadrozole (PDB ligand ID: 0T3),^51^ which are potent inhibitors reported in crystallographic complexes with CYP11B1 and CYP11B2, respectively. Conversely, no significant ligand similarity was observed according to the MACCS fingerprints (data not shown).

**Table 1:** Number of Synthesized Compounds Showing Similarity Values Above Commonly Accepted Thresholds,^52^ with Respect to Molecules with Activity Annotations on CYP19A1, CYP11B1, and CYP11B2^a^

compound ID	CYP19A		CYP11B1		CYP11B2	
	*N* similar compounds based on ECFP4*fp*^b^	*N* similar compounds based on TanimotoCombo^c^	*N* similar compounds based on ECFP4*fp*^b^	*N* similar compounds based on TanimotoCombo^c^	*N* similar compounds based on ECFP4*fp*^b^	*N* similar compounds based on TanimotoCombo^c^
**X1**	0, 2	0, 0, 0	0, 2	0, 0, 4	1, 1	0, 0, 4
**X2**	0, 3	0, 0, 0	0, 3	0, 0, 15	3, 2	0, 0, 14
**X3**	0, 3	0, 0, 5	0, 3	0, 0, 24	3, 2	0, 0, 21
**X4**	0, 1	0, 0, 0	0, 1	0, 0, 0	0, 0	0, 0, 0
**X5**	0, 0	0, 0, 0	0, 1	0, 0, 4	0, 0	0, 0, 4
**X6**	0, 3	0, 0, 0	0, 3	0, 0, 17	3, 2	0, 0, 16
**X7**	0, 1	0, 0, 0	0, 1	0, 0, 3	0, 0	0, 0, 2
**X8**	0, 3	0, 0, 0	0, 2	0, 0, 7	1, 1	0, 0, 6
**X9**	0, 1	0, 0, 0	0, 0	0, 0, 0	0, 0	0, 0, 0
**X10**	0, 1	0, 0, 0	0, 0	0, 0, 1	0, 0	0, 0, 1
**X11**	0, 1	0, 0, 0	0, 1	0, 0, 0	0, 0	0, 0, 0
**X12**	0, 1	0, 0, 0	0, 1	0, 0, 0	0, 0	0, 0, 0
**X13**	0, 2	0, 0, 0	0, 1	0, 0, 0	2, 0	0, 0, 0
**X14**	0, 2	0, 0, 0	0, 1	0, 0, 0	0, 1	0, 0, 0
**X15**	0, 1	0, 0, 0	0, 1	0, 0, 0	0, 0	0, 0, 0
**X16**	0, 1	0, 0, 0	0, 0	0, 0, 0	0, 0	0, 0, 0
**X17**	0, 1	0, 0, 0	0, 1	0, 0, 0	0, 0	0, 0, 0
**X18**	0, 1	0, 0, 0	0, 1	0, 0, 3	0, 0	0, 0, 3
**X19**	0, 1	0, 0, 0	0, 1	0, 0, 3	0, 0	0, 0, 3
**X20**	0, 1	0, 0, 3	1, 1	0, 0, 20	2, 0	0, 0, 18
**X21**	0, 2	0, 0, 7	1, 1	1, 1, 15	3, 1	1, 1, 15
**X22**	0, 2	0, 0, 0	1, 2	0, 0, 0	5, 2	0, 0, 0
**X23**	0, 2	0, 0, 0	0, 1	0, 0, 3	0, 0	0, 0, 2
**X24**	0, 1	0, 0, 2	1, 1	0, 0, 9	2, 0	0, 0, 5

a ECFP4*fp*-based similarity estimations were performed with the LigAdvisor Web server,^25^ while 3D similarity estimations were made with the ROCS software.^53^

b For each synthesized compound, the number of “PDB ligands, DrugBank ligands” that showed Tanimoto index above 0.3 is reported.

c For each synthesized compound, the number of “PDB ligands, DrugBank ligands, ChEMBL ligands” that showed TanimotoCombo index above 1.5 is reported (only ChEMBL ligands with reported IC_50_, *K*_i_, *K*_d_, EC_50_, and potency below 1 μM were taken into consideration).

**Figure 3:**
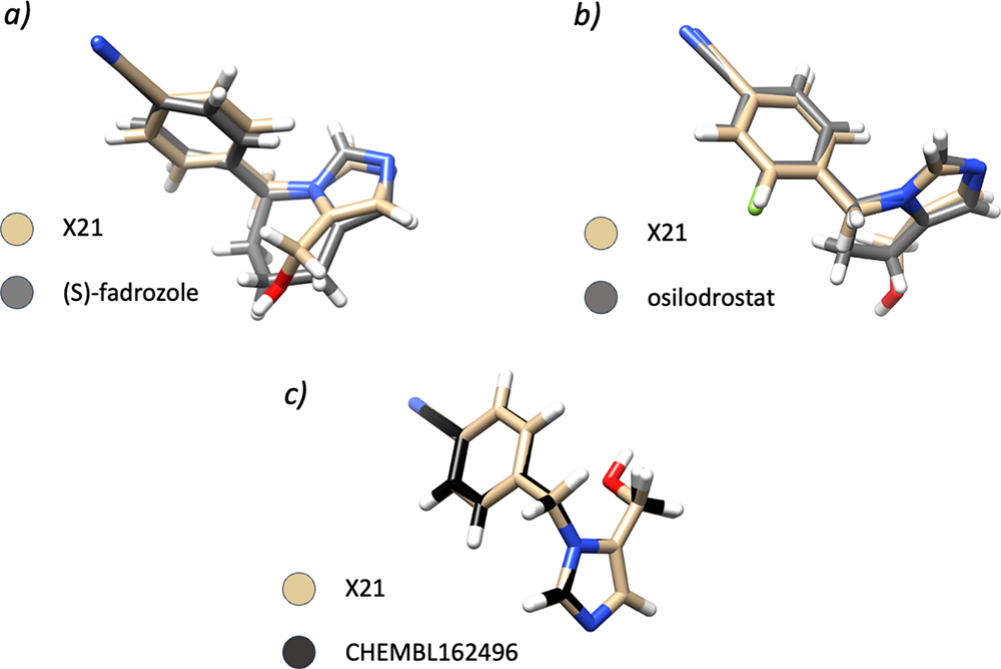
Predicted 3D ROCS-based alignments of compound **X21** with (*S*)-fadrozole (a), osilodrostat (b), and CHEMBL162496 (c).

A series of 3D similarity evaluations with respect to ligands extracted from DrugBank, PDB, and ChEMBL^54,55^ were also performed by using ROCS, as detailed in Methods section. 3D-similarity evaluations revealed that compound **X21** was the only one to have a significant (*TanimotoCombo* index higher than 1.5) steric and electrostatic overlap with (*S*)*-*fadrozole (PDB ligand ID: JD7) (Figure 3a) and (*R*)-fadrozole (PDB ligand ID: 0T3), which have been cocrystallized with CYP11B2 (PDB ID: 6M7X)^49^ and CYP11B1 (PDB ID: 4FDH),^51^ respectively (Table S3). Notably, fadrozole has already been tested in vitro against CYP19A1,^56,57^ CYP11B1,^32−34^ and CYP11B2^33^ with good outcomes, supporting the selection of compound **X21** as a valuable candidate for further evaluation. However, it should be pointed out that fadrozole lacks selectivity against CYP11B1.^32−34^ Similar results arose from the 3D-similarity analyses against DrugBank compounds (Table S3), which allowed the identification of a notable degree of similarity between compound **X21** and DB11837 (osilodrostat, PDB ligand ID: YSY) (Figure 3b), the latter compound being active against all investigated CYP19A1, CYP11B1, and CYP11B2 enzymes.^32,33^ Finally, 3D similarities against ChEMBL ligands allowed the identification of four similar CYP19A1 inhibitors, 14 CYP11B1 inhibitors, and 14 CYP11B2 inhibitors (Table S2).

**Figure 4:**
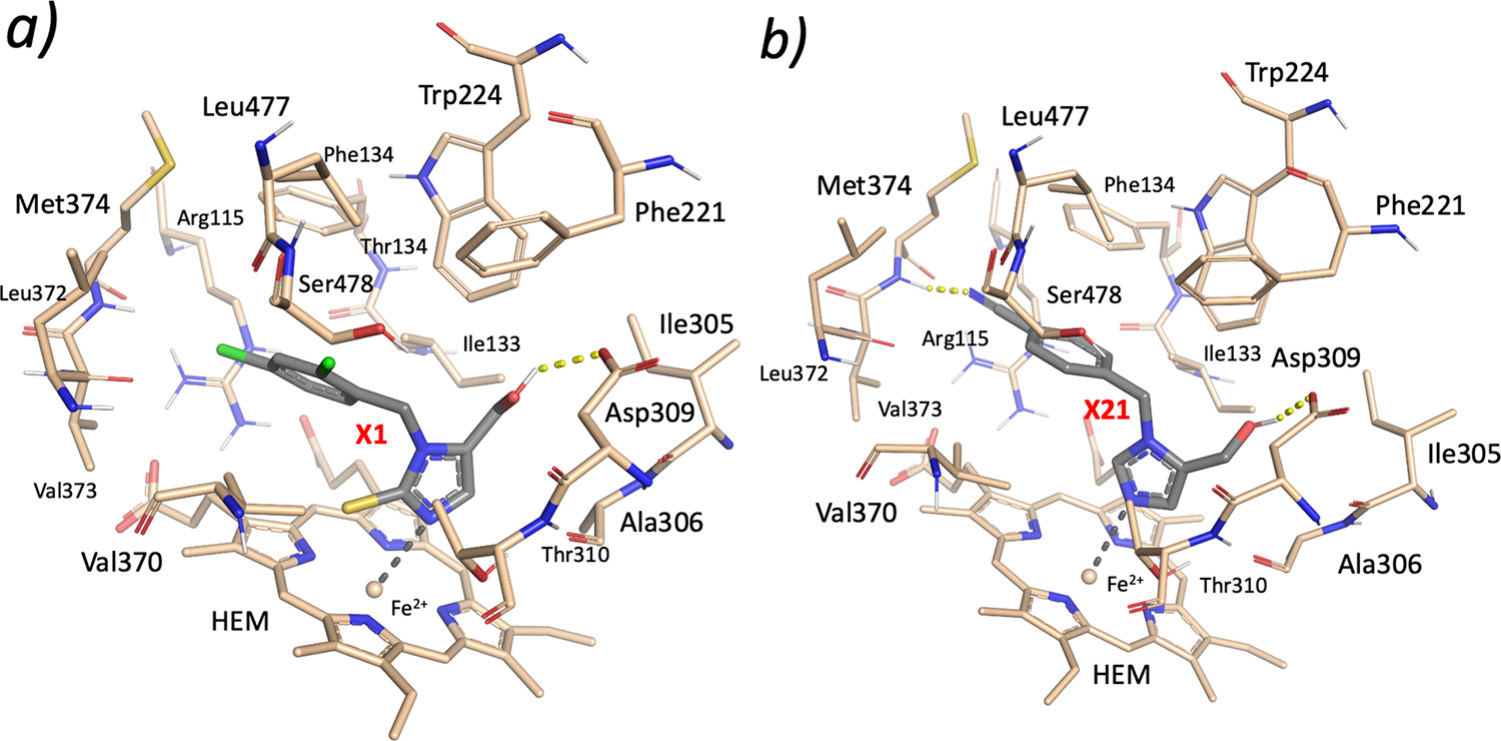
Predicted binding mode of compounds **X1** (a) and **X21** (b) into the aromatase (CYP19A1) binding site.

Again, compound **X21** emerged as the top-ranking candidate, being the only one to show a good overlap with aromatase ligands reported in ChEMBL (e.g., CHEMBL162496, IC_50_ of 15 nM)^28^ (Figure 3c). Importantly, compound **X21** was previously reported to be active against aldosterone synthase (CYP11B2, IC_50_ = 29 nM) and to be 10-fold less active against 11β-hydroxylase (CYP11B1, IC_50_ = 285 nM),^33^ but to the best of our knowledge it was never tested against aromatase. The ligand-based analyses described above were complemented with structure-based analyses, e.g., docking into the binding sites of CYP19A1, CYP11B1, and CYP11B2 by means of FRED (OpenEye).^58^ According to docking, only compounds **X1** and **X21** showed good complementarity with aromatase (Figure 4a,b), the predicted binding scores (Table S3) being better than those of (*R*)- and (*S*)*-*fadrozole.

In the predicted docking poses (Figure 4), the hydroxyl group of the two compounds H-bonds with Asp309, while the imidazole nitrogen lone pair coordinates the Fe^2+^ ion of HEME, similarly to (*R*)- and (*S*)-fadrozole (Figure S1a,b). Moreover, compound **X21** makes an additional hydrogen bond with the backbone of Met374 (Figure 4b), thus mimicking the H-bond established by the carbonyl group of androst-4-ene-3,17-dione (PDB ligand ID: ASD).^59^ Docking calculations of **X21** into the CYP11B1 binding site showed that the compound hydrogen bonds with the backbone atoms of Leu382 and Ala313, and coordinates the Fe^2+^ ion of HEME. A similar binding pose was obtained in CYP11B2; but in this case, the hydroxyl group of the ligand was not engaged in hydrogen bonds with active site residues (Figure S1d).

### Biological Evaluation

#### In Vitro Inhibitory Activity

Standing on the results described above, compounds **X1** and **X21** emerged as the most promising compounds for biological testing. Hence, the two compounds were tested in vitro to assess their inhibitory activity against the recombinant aromatase enzyme, using letrozole as a reference (Table 2).^60^

**Table 2:** Inhibitory Activity of **X21** (IC_50_, nM) against the CYP19A1 (Aromatase), CYP11B2 (Aldosterone Synthase), and CYP11B1 (11β-Hydroxylase) Enzymes

compound	IC_50_ (nM)			CYP11B1/CYP11B2 selectivity ratio
	CYP19A1	CYP11B2	CYP11B1	
**X21**	2.3	29^a^	285^a^	9.8
**(*S*)-fadrozole**	3.0–17^b^	171^c^	40^c^	0.2
**(*R*)-fadrozole**	680–6000^d^^,^^e^	6.0^c^	11^c^	1.8
**letrozole**	0.5	1420^f^	2620^f^	1.8

a Note: ref (33).

b Refs (56,57).

c Refs (32−34).

d Ref (56).

e Ref (61).

f Ref (60).

Unfortunately, compound **X1** showed no inhibition of CYP19A1. To find an explanation, we hypothesized that the lack of activity could be due to the tautomeric equilibria of the thiol group present on the imidazole ring. Quantum mechanical calculations made with Jaguar^62^ confirmed that the thioketone form was several kcals/mol more stable than the thiol form (data not shown). In the thioketone tautomer, the coordination of the Fe^2+^ of the HEME group by means of the imidazole nitrogen lone pair would be disrupted, thus explaining the observed lack of activity. Gratifyingly, compound **X21** displayed potent nanomolar inhibitory activity of aromatase (CYP19A1, IC_50_ of 2.3 nM, Table 2), which adds to the already reported potent and selective inhibition of aldosterone synthase (CYP11B2, IC_50_ of 29 nM)^33^ and 10-fold selectivity with respect to 11β-hydroxylase (CYP11B1, IC_50_ of 285 nM). The excellent inhibitory activity and selectivity of compound **X21** are likely due to the presence of the hydroxymethyl group, which hydrogen bonds to the side chain of Asp309 in CYP19A1 but not in CYP11B2, where it is placed into a small lipophilic pocket.

To further characterize the potential effects of compound **X21** on metabolic stability, in vitro assays against cytochromes P450 1A2 (CYP1A2) and 3A4 (CYP3A4) were also conducted. The latter enzymes were selected among those normally expressed in cells due to their major roles in the oxidation of xenobiotics (e.g., toxins and drugs).^63^ Importantly, these assays revealed that compound **X21** had marginal inhibitory activity of these enzymes, with the IC_50_ values being higher than 50 μM (Figure S2).

**Figure 5:**
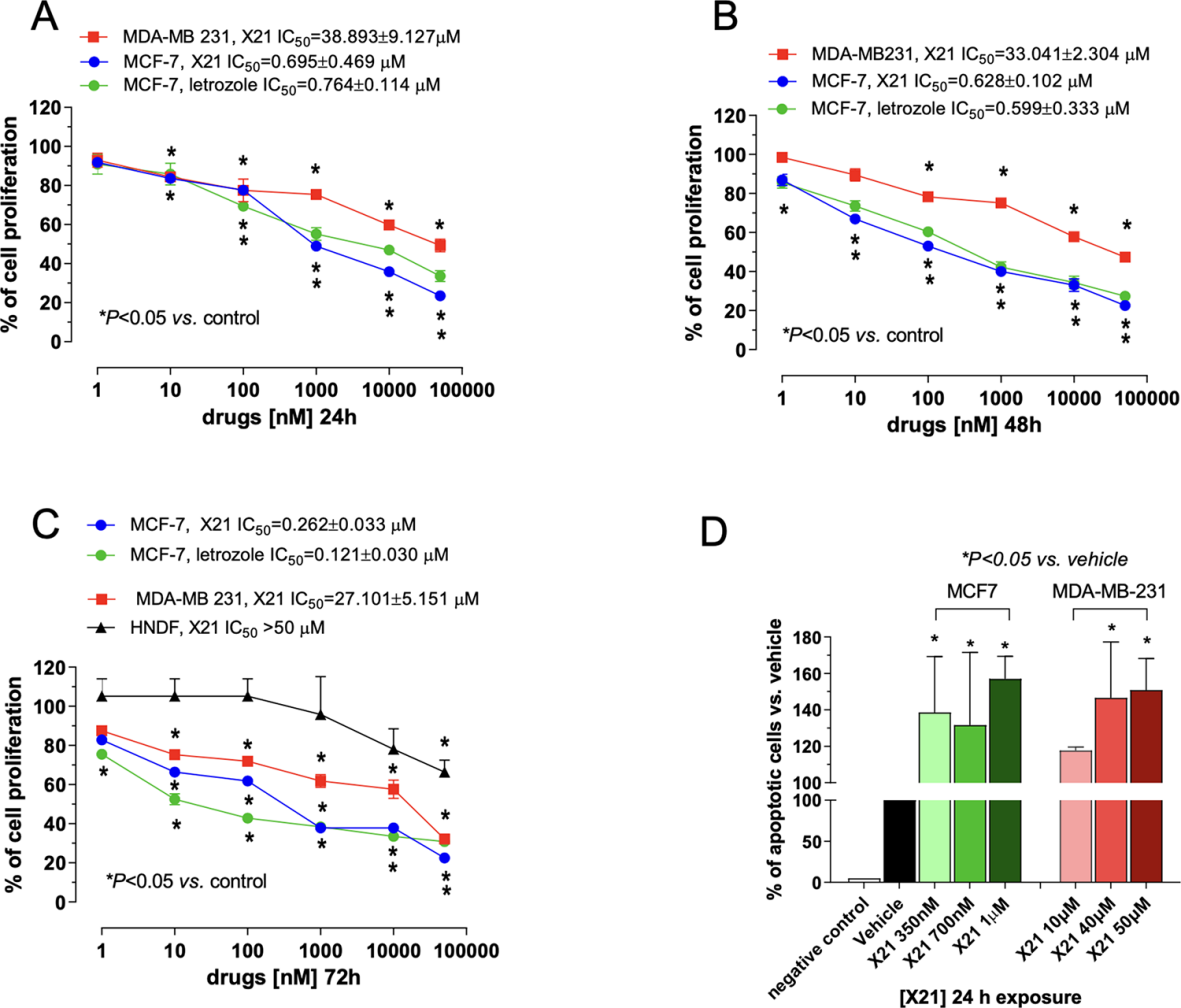
Antiproliferative in vitro effects of compound **X21** and letrozole on human MCF-7 (Estrogen Receptor+), MDA-MB-231 (Estrogen Receptor−), and HNDF healthy cells at 24 h (A), 48 h (B), and 72 h (C). The data are presented as mean (±SEM) percentage values of vehicle-treated cell proliferation. Pro-apoptotic effects were observed in MCF-7 and MDA-MB-231 cells (D) using the cell death detection ELISA Plus kit. The internal negative control was provided by an ELISA kit. Columns and bars, mean values ± SD, respectively.

Finally, the benzylimidazole **X21** was evaluated for its ability to cause antiproliferative and pro-apoptotic activity on two human BC cell lines (MCF-7, ER and PR positive, and MDA-MB-231, ER and PR negative) and one human normal dermal human fibroblast cell line (HNDF). The antiproliferative parameters, expressed in terms of IC_50_ values obtained after 24, 48, and 72 h of drug-exposure, are shown in Figure 5.

Compound **X21** showed a time- and concentration-dependent proliferation inhibition on both tested cancer cell lines (Figure 5). However, marked differences of potency were found between the two cell lines, the MCF-7 resulting in being the most sensitive cell line to compound **X21** compared to MDA-MB-231 (Figure 5A–C). In particular, at 72 h, compound **X21** inhibited the MCF-7 cell proliferation with an IC_50_ value of 0.26 ± 0.03 μM, whereas the antiproliferative activity on MDA-MB-231 was much lower (IC_50_ = 27.10 ± 5.15 μM; Figure 5C). Interestingly, compound **X21** showed a similar activity to that of letrozole used as a reference (IC_50_ = 0.12 ± 0.03 μM; Figure 5C), as well as higher antiproliferative activity with respect to fadrozole (see Figure S3 in the Supporting Information). Remarkably, no significant antiproliferative effect on HNDF was found at the tested drug concentrations except for 50 μM (a 30% inhibition compared to vehicle; Figure 5C), thus confirming the lack of toxicity on normal cells. The apoptotic process was quantified using an ELISA test. Figure 5D shows a significant increase in the extent of DNA fragmentation at nanomolar concentrations of **X21** after 24 h of exposure in MCF-7 cancer cells compared to vehicle-treated cells, whereas only higher concentrations (>40 μM) of compound **X21** significantly increased the apoptotic signal in MDA-MB-231 cells (Figure 5D).

**Figure 6:**
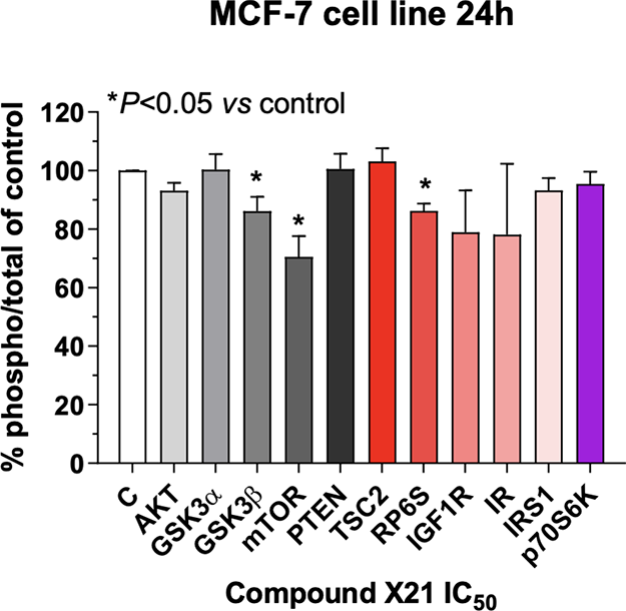
Luminex analysis of the Akt/mTOR cell signaling pathway in MCF-7 cells treated with compound **X21** for 24 h at the experimental antiproliferative IC_50_ (700 nM). Results were reported as the percentage of the phosphorylated protein/total protein ratio vs 100% of vehicle-treated cells. C, vehicle-treated control; AKT, protein kinase B; GSK, glycogen synthase kinase 3; mTOR, mammalian target of rapamycin; PTEN, phosphatase and tensin homologue; TSC2, tuberous sclerosis complex 2; RP6S, ribosomal protein S6; IGF1R, insulin-like growth factor 1 (IGF-1) receptor; IR, insulin receptor; IRS1, insulin receptor substrate 1; p70S6K, ribosomal protein S6 kinase beta-1. Columns and bars, mean values ± SD, respectively.

Searching for other molecular mechanisms underlying the pharmacological activity exhibited by **X21**, the ability of this compound to inhibit the phosphorylation of enzymes involved in the Akt/mTOR cell signaling pathway was investigated by luminex analysis of cell lysates. The activation of mTOR signaling in BC cells is associated with resistance to multiple drug therapies because the PI3K/Akt/mTOR axis represents an escape for the dependency from ER signaling. Indeed, the inhibition of mTOR has been shown to resensitize cells to the effects of tamoxifen.^64^ Compound **X21** was tested for its ability to inhibit protein phosphorylation in the MCF-7 cell line after 24 h exposure. As shown in Figure 6, the compound significantly inhibited mTOR phosphorylation (−30% vs control) at a concentration corresponding to its experimental IC_50_. A lower, but still significant, inhibition was also found in the phosphorylation of GSK3α and RP6S enzymes (Figure 6).

Finally, in order to evaluate potential cardiovascular issues arising from the administration of compound **X21**, the compound was tested for its ability to interfere with the human cardiac potassium and sodium channels. To this aim, *h*ERG and Nav1.5 manual patch clamp assays were conducted as described in the Methods section. Titration curves of compound **X21** and the reference compounds E-4031 and tetrodoxin are reported in Figure S4. Satisfyingly, no significant inhibition of *h*ERG and Nav1.5 was observed up to 10 and 30 μM concentrations, respectively, suggesting that compound **X21** is potentially safe with respect to cardiotoxicity issues. As for the importance of balanced aldosterone levels in cardiac safety, the potent inhibition of aldosterone synthase CYP11B2 exerted by **X21** is a major determinant of cardiac safety, which is inherent to the mechanism of action of this drug.^8,24^

## Conclusions

In this study, we describe the synthesis and computational analysis of a library of variously decorated benzylimidazole derivatives. The best candidates were biologically tested in an effort toward identifying potent and safe dual inhibitors of aromatase and aldosterone synthase. To this aim, 24 compounds with a benzylimidazole scaffold bearing different structural decorations were synthesized. The compounds were investigated by means of 2D- and 3D-similarity estimations made with LigAdvisor^25^ and ROCS,^53^ complemented by docking analyses into the investigated target enzymes, resulting in the selection of two candidates that were in vitro tested, namely, compounds **X1** and **X21**. Compound **X21** showed the desired, potent, and balanced dual inhibition of aromatase and aldosterone synthase and >10-fold selectivity with respect to 11β-hydroxylase, thus emerging as the best candidate. As such, compound **X21** was further evaluated to assess its activity against additional selected cytochrome P450 enzymes responsible for metabolism of xenobiotics. The compound showed excellent antiproliferative and pro-apoptotic activity against the ER+ MCF-7 cell line. Of note, the same antiproliferative and apoptotic effects in ER-negative MDA-MB-231 cells were obtained at very high concentrations and were almost absent in normal human fibroblasts. Interestingly, compound **X21** also showed characteristics to significantly inhibit the phosphorylation of mTOR in MCF-7 cells. Importantly, the compound showed negligible cardiotoxicity as assessed by *h*ERG and Nav1.5 inhibition assays. Therefore, compound **X21** stands out as a very interesting and promising candidate for further evaluation.

## Experimental Section

### Chemistry

#### General Information

Unless specified, reagents were obtained from commercial sources and used without further purification. Solvents were obtained from Fischer Scientific. Melting points were recorded on an Optimelt automated melting point system and are uncorrected. The heating ramp gradient was set at 2.5 °C min^–1^. Flash chromatography was performed using Merck Silica gel high-purity grade (9385), pore size 60 Å, 230–400 mesh particle size. Thin-layer chromatography was performed using Merck TLC silica gel 60 with glass support. IR spectra were recorded neatly on a PerkinElmer Spectrum Two FT-IR spectrometer. The absorbency of the peaks was defined as weak (w, <40% of most intense peak), medium (m, 40–75% of the most intense peak), strong (s, >75% of the most intense peak), and broad (br). Nuclear magnetic resonance (NMR) spectra were recorded on a Bruker Avance III HD 400 spectrometer with operating frequencies of 400 MHz for 1H, 101 MHz for ^13^C. Proton chemical shift values are given in units δ relative to residual protic solvent. The multiplicity of the signal is indicated as br—broad, s—singlet, d—doublet, t—triplet, q—quartet, and m—multiplet, dd—doublet of doublets, dt—doublet of triplets, etc. Coupling constants (*J*) were measured to the nearest 0.1 Hz. Carbon chemical shift data are given in units δ relative to residual protic solvent. 2D NMR was used to aid the assignment of signal in ^13^C NMR. Liquid chromatography–mass spectrometry (LC-MS) was performed on a TQD mass spectrometer and an Acquity UPLC (Waters Ltd., UK).

#### Experimental Preparation for Compounds **X4**, **X11–12**, and **X14–18**

In a typical reaction, based upon 1 mmol of the free thiol; 2 equiv of the acceptor and 2 equiv of triethylamine were dissolved in a 1:1 mixture of DMSO:MeCN (2 mL each), the thiol was added, and the mixture was stirred at 90 °C for 2–4 h while monitoring by (EtOAc:hexane, 6:4). Upon completion, the reaction mixture was poured into water and the resulting solid filtered or extracted with EtOAc (2 × 25 mL), dried over MgSO_4_ and evaporated to dryness. Purification was performed by trituration, crystallization, or column chromatography as indicated.

#### (1-(Benzo[*d*][1,3]dioxol-5-ylmethyl)-2-((2-nitro-5-(trifluoromethyl)phenyl)thio)-1*H*-imidazol-5-yl)methanol, Compound **X17**

Chemical formula: C_19_H_14_F_3_N_3_O_5_S.

Pale yellow solid isolated in 84% by crystallization from EtOAc:hexane 1:3; melting point 211.6–213.8 °C. LC-MS *R*_t_ 2.37 min *m*/*z* = 454.19 MeCN; HRMS calculated for C_19_H_15_N_3_O_5_S as 454.0679, found 454.0682 (Δ = 0.7 ppm); ^1^H NMR (400 MHz, DMSO-*d*_6_) δ 8.37 (d, *J* = 2.1 Hz, 1H), 7.76 (dd, *J* = 8.6, 2.1 Hz, 1H), 7.30 (s, 1H), 6.63 (d, *J* = 8.6 Hz, 1H), 6.57 (d, *J* = 1.7 Hz, 1H), 6.53–6.43 (m, 2H), 5.84 (s, 2H), 5.52 (t, *J* = 5.1 Hz, 1H), 5.18 (s, 2H), 4.57 (br. s, 2H); ^13^C NMR (101 MHz, DMSO-*d*_6_) δ 147.40 (C), 146.65 (C), 144.83 (C), 141.13 (C), 137.80 (C), 134.64 (C), 130.48 (CH), 130.35 (q, *J* = 3.3 Hz, CH), 130.24 (C), 129.64 (CH), 126.99 (q, *J* = 33.8 Hz, C), 123.30 (q, *J* = 271.7 Hz, C), 23.19 (q, *J* = 4.1 Hz, CH), 121.15 (CH), 108.24 (2 × CH), 101.40 (CH_2_), 53.72 (CH_2_), 48.16 (CH_2_); ^19^F NMR (376 MHz, DMSO-*d*_6_) δ −61.41; IR ν = 3133 br. w, 2909 br. w, 1622 w, 1567 w, 1528 m, 1492 m, 1446 m, 1422 m, 1326 s, 1301 s, 1247 s, 1152 s, 1121 s, 1031 s, 944 w, 925 m cm^–1^. *The signals for CH carbons correlating with the 1,3-benzodioxole ring signals at 6.55 and 6.48 appear coincident, as proven by HR-NMR and HSQC/HMBC 2D spectroscopy.

^1^H NMR (599 MHz, DMSO-*d*6) δ 8.35 (d, *J* = 2.1 Hz, 1H), 7.74 (dd, *J* = 8.6, 2.1 Hz, 1H), 7.27 (s, 1H), 6.61 (d, *J* = 8.6 Hz, 1H), 6.55 (d, *J* = 1.6 Hz, 1H), 6.48 (d, *J* = 7.9 Hz, 1H), 6.45 (dd, *J* = 7.9, 1.6 Hz, 1H), 5.82 (s, 2H), 5.49 (app t., *J* = 5.2 Hz, 1H), 5.16 (s, 2H), 4.55 (br. s, *J* = 3.4 Hz, 2H); ^13^C NMR (151 MHz, DMSO-*d*6) δ 147.39 (C), 146.64 (C), 144.82 (C), 141.10 (C), 137.78 (C), 134.63 (C), 130.46 (CH), 130.32 (q, *J* = 3.3 Hz, CH), 130.22 (C), 129.63 (CH), 126.99 (q, *J* = 34.0 Hz, C), 123.36 (q, *J* = 271.7 Hz, C), 123.15 (q, *J* = 4.1 Hz, CH), 121.32 (CH), 108.27 (CH), 108.22 (CH), 101.38 (CH_2_), 53.71 (CH_2_), 48.14 (CH_2_).

#### (2-((5-Chloro-2-nitrophenyl)thio)-1-(2-(thiophen-2-yl)ethyl)-1*H*-imidazol-5-yl)methanol, Compound **X14**

Chemical formula: C_16_H_14_ClN_3_O_3_S_2_.

Pale yellow solid isolated by trituration with 2:8 EtOAc:hexane in 73% yield; melting point 150.6–153.5 °C. LC-MS *R*_t_ 2.24 min *m*/*z* = 396.16 MeCN; HRMS calculated for C_16_H_15_^35^ClN_3_O_3_S_2_ as 396.0238, found 396.0244 (Δ = 1.5 ppm); ^1^H NMR (400 MHz, DMSO-*d*_6_) δ 8.29 (d, *J* = 8.9 Hz, 1H), 7.51 (dd, *J* = 8.9, 2.2 Hz, 1H), 7.29 (dd, *J* = 5.1, 1.2 Hz, 1H), 7.24 (s, 1H), 6.86 (dd, *J* = 5.1, 3.4 Hz, 1H), 6.68 (dd, *J* = 3.4, 1.2 Hz, 1H), 6.60 (d, *J* = 2.2 Hz, 1H), 5.41 (t, *J* = 5.3 Hz, 1H), 4.42 (d, *J* = 5.3 Hz, 2H), 4.32 (t, *J* = 7.0 Hz, 2H), 3.14 (t, *J* = 7.0 Hz, 2H); ^13^C NMR (101 MHz, DMSO-*d*_6_) δ 143.72 (C), 140.20 (C), 139.64 (C), 138.84 (C), 137.30 (C), 134.84 (C), 130.55 (CH), 128.49 (CH), 127.50 (CH), 127.22 (CH), 127.09 (CH), 126.50 (CH), 125.30 (CH), 53.62 (CH_2_), 46.66 (CH_2_), 30.73 (CH_2_); IR ν = 3164 br. w, 2905 br. w, 1622 w, 1584 w, 1554 w, 1505 m, 1445 m, 1419 m, 1330 s, 1301 m, 1229 m, 1149 w, 1137 m, 1088 w, 1029 s, 925 m cm^–1^.

#### (1-Benzyl-2-((2-nitro-4-(trifluoromethyl)phenyl)thio)-1*H*-imidazol-5-yl)methanol, Compound **X12**

Chemical formula: C_18_H_14_F_3_N_3_O_3_S.

Off-white solid isolated in 82% yield crystallized from MeOH:DCM 1:15; melting point 225.4–226.9 °C. LC-MS *R*_t_ 2.47 min *m*/*z* = 410.22; HRMS calculated for C_18_H_15_F_3_N_3_O_3_S as 410.0781, found 410.0770 (Δ = −2.7 ppm); ^1^H NMR (400 MHz, DMSO-*d*_6_) δ 8.35 (d, *J* = 2.1 Hz, 1H), 7.77 (dd, *J* = 8.6, 2.1 Hz, 1H), 7.32 (s, 1H), 7.13–7.01 (m, 3H), 7.00–6.94 (m, 2H), 6.70 (d, *J* = 8.6 Hz, 1H), 5.46 (t, *J* = 5.2 Hz, 1H), 5.30 (s, 2H), 4.54 (d, *J* = 5.2 Hz, 2H); ^13^C NMR (101 MHz, DMSO-*d*_6_) δ 144.85 (C), 141.09 (C), 137.85 (C), 136.53 (CH), 134.83 (C), 130.66 (q, *J* = 3.4 Hz, CH), 130.55 (C), 129.60 (CH), 128.76 (2 × CH), 127.59 (CH), 127.30 (2 × CH), 127.05 (q, *J* = 33.8 Hz, C), 123.34 (q, *J* = 271.9 Hz, C), 123. 31 (q, *J* = 4.1 Hz, CH), 53.88 (CH_2_), 48.29 (CH_2_); ^19^F NMR (376 MHz, DMSO-*d*_6_) δ −61.41; IR ν = 3116 br. w, 2775 br. w, 1621 w, 1567 w, 1530 m, 1457 w, 1426 m, 1352 m, 1326 s, 1254 m, 1149 m, 1124 s, 1077 m, 1037 m, 905 m cm^–1^.

#### (1-Benzyl-2-((2-nitro-5-(trifluoromethyl)phenyl)thio)-1*H*-imidazol-5-yl)methanol, Compound **X11**

Chemical formula: C_18_H_14_F_3_N_3_O_3_S.

Pale yellow solid isolated in 77% yield by crystallization from 8:2 EtOAc:hexane, melting point 222.4–223.8 °C. LC-MS *R*_t_ 2.40 min *m*/*z* = 410.18; HRMS calculated for C_18_H_15_F_3_N_3_O_3_S as 410.0781, found 410.0789 (Δ = 2.0 ppm); ^1^H NMR (400 MHz, DMSO-*d*_6_) δ 8.25 (dd, *J* = 8.6, 1.0 Hz, 1H), 7.66 (dd, *J* = 8.6, 1.9 Hz, 1H), 7.34 (s, 1H), 7.03–6.94 (m, 5H), 6.74 (d, *J* = 1.9 Hz, 1H), 5.49 (br s, 1H), 5.34 (s, 2H), 4.56 (br s, 2H); ^13^C NMR (101 MHz, DMSO-*d*_6_) δ 147.13 (C), 137.94 (C), 137.26 (C), 136.48 (C), 134.88 (C), 133.49 (q, *J* = 31.1 Hz, C), 130.49 (CH), 128.63 (CH), 127.64 (CH), 127.59 (CH), 127.29 (CH), 125.04 (q, *J* = 4.5 Hz, CH), 123.77 (q, *J* = 3.4 Hz, CH), 123.17 (q, *J* = 274.8 Hz, C), 53.81 (CH_2_), 48.37 (CH_2_); ^19^F NMR (376 MHz, DMSO-*d*_6_) δ −62.42; IR ν = 3133 br w, 2770 br w, 1641 w, 1536 m, 1465 w, 1430 m, 1418 w, 1350 s, 1330 s, 1253 m, 1155 s, 1128 s, 10827 s, 1035 m, 899 m cm^–1^.

#### (1-Benzyl-2-(methylthio)-1*H*-imidazol-5-yl)methanol, Compound **X18**

Chemical formula: C_12_H_14_N_2_OS.

White solid, isolated in 79% yield by column chromatography using EtOAc:hexane 7:3; melting point 104.7–106.8 °C (lit. m.p. 103–105 °C EtOAc).^65^ LC-MS Rt 0.814 min *m*/*z* 235.18; HRMS calculated for C_12_H_15_N_2_OS as 235.0900, found 235.0895 (Δ = −2.1 ppm); ^1^H NMR (400 MHz, DMSO-*d*_6_) δ 7.38–7.31 (m, 2H), 7.30–7.23 (m, 1H), 7.13–7.04 (m, 2H), 6.95 (s, 1H), 5.23 (app. t, *J* = 5.0 Hz, 3H), 4.35 (d, *J* = 5.0 Hz, 2H), 2.45 (s, 3H); ^13^C NMR (101 MHz, DMSO-*d*_6_) δ 143.23 (C), 137.50 (C), 134.40 (C), 129.06 (2 × CH), 128.13 (CH), 127.84 (CH), 126.89 (2 × CH), 53.61 (CH_2_), 47.30 (CH_2_), 16.25 (CH_3_); IR ν = 3117 br w, 2839 br w, 2738 br w, 1604 w, 1501 m, 1445 s, 1415 s, 1372 m, 1308 s, 1279 m, 1183 w, 1141 m, 1082 w, 1016 s, 975 m, 914 w cm^–1^.

#### (1-Benzyl-2-((5-chloro-2-nitrophenyl)thio)-1*H*-imidazol-5-yl)methanol, Compound **X4**

Chemical formula: C_17_H_14_ClN_3_O_3_S.

Yellow solid isolated in 76% using trituration with EtOAc:Hexane 2:10, melting point 186–188.5 °C. LC-MS R_t_ 2.76 min *m*/*z* = 376.21 MeCN; HRMS calculated for C_17_H_15_^35^ClN_3_O_3_S as 376.0517, found 376.0519 (Δ = 0.5 ppm); ^1^H NMR (400 MHz, DMSO-*d*_6_) δ 8.17 (d, *J* = 2.3 Hz, 1H), 7.54 (dd, *J* = 8.8, 2.3 Hz, 1H), 7.29 (s, 1H), 7.17–7.10 (m, 3H), 7.00–6.94 (m, 2H), 6.56 (d, *J* = 8.8 Hz, 1H), 5.44 (br s, 1H), 5.29 (s, 2H), 4.50 (s, 2H); ^13^C NMR (101 MHz, DMSO) δ 145.41 (C), 137.62 (C), 136.66 (C), 135.34 (C), 134.70 (C), 134.54 (CH), 131.08 (C), 130.40 (CH), 129.82 (CH), 128.84 (2 × CH), 127.60 (CH), 127.16 (2 × CH), 125.75 (CH), 53.90 (CH_2_), 48.22 (CH_2_); IR ν = 3127 br w, 1655 w, 1590 m, 1561 w, 1514 m, 1455 m, 1420 m, 1331 s, 1305 s, 1124 m, 1038 m, 1000 w, 860 m, 831 m cm^–1^.

#### (1-Benzyl-2-((3,5-dichloro-2,6-difluoropyridin-4-yl)thio)-1*H*-imidazol-5-yl)methanol, Compound **X15**

Chemical formula: C_16_H_11_Cl_2_F_2_N_3_OS.

Pale tan solid isolated in 34% yield as a mixture with **X16** following separation using column chromatography with EtOAc:hexane 8:2; melting point 191.0 °C (decompose). LC-MS (MeCN) *R*_t_ 2.64 min *m*/*z* = 421.18; HRMS calculated for C_16_H_12_^35^Cl_2_F_2_N_3_OS as 421.0025, found 421.0021 (Δ = −1.0 ppm); ^1^H NMR (400 MHz, DMSO-*d*_6_) δ 7.33–7.17 (m, 3H), 7.11 (s, 1H), 7.03–6.93 (m, 2H), 5.43–5.36 (m, 3H), 4.44 (d, *J* = 4.8 Hz, 2H); ^13^C NMR (101 MHz, DMSO-*d*_6_) δ 156.46 (C), 154.06 (C), 147.57 (C), 144.84 (dd, *J* = 220.1, 14.8 Hz, C), 129.76 (CH), 129.43 (d, *J* = 5.9 Hz, C), 128.86 (2 × CH), 128.05 (CH), 126.28 (2 × CH), 118.88 (d, *J* = 34.6 Hz, C), 53.68 (CH_2_), 48.14 (CH_2_); 19F NMR (376 MHz, DMSO-*d*_6_) δ −70.02; IR ν = 3183 br. w, 2958 br. w, 1654 br. m, 1543 m, 1496 w, 1450 m, 1421 m, 1355 s, 1334 s, 1253 w, 1145 w, 1096 w, 1034 s, 1028 m, 833 s cm^–1^.

#### 1-Benzyl-2-((3,5-dichloro-2,6-difluoropyridin-4-yl)thio)-1*H*-imidazole-5-carbaldehyde, Compound **X16**

Chemical formula: C_16_H_9_Cl_2_F_2_N_3_OS.

Off white solid generated by air oxidation of compound **X15** in 56% yield following separation using column chromatography with EtOAc:hexane 8:2; melting point 107.8–110.9 °C. LC-MS *R*_t_ 2.87 min *m*/*z* = 400.15 and 402.13 MeCN; HRMS calculated for C_16_H_10_Cl_2_F_2_N_3_OS as 417.9790, found 417.9783 (Δ = −1.7 ppm); ^1^H NMR (400 MHz, DMSO-*d*_6_) δ 9.75 (s, 1H), 8.06 (s, 1H), 7.45–7.21 (m, 3H), 7.18–7.05 (m, 2H), 5.69 (s, 2H); ^13^C NMR (101 MHz, DMSO-*d*_6_) δ 180.50 (CH), 154.45 (dd, *J* = 246.2, 15.3 Hz, C), 147.35 (t, *J* = 1.5 Hz, C), 145.07 (C), 144.23 (C), 135.93 (C), 133.47 (C), 129.17 (2 × CH), 128.38 (CH), 126.91 (2 × CH), 117.78 (d, *J* = 40.2 Hz, C), 49.52 (CH_2_); ^19^F NMR (376 MHz, DMSO-*d*_6_) δ −70.77; IR ν = 3085 br w, 1667 s, 1577 s, 1528 w, 1495 w, 1445 m, 1397 m, 1393 s, 1336 m, 1273 w, 1246 w, 1099 w, 1029 w, 938 w, 881 s cm^–1^.

#### 4-((5-(Hydroxymethyl)-1*H*-imidazol-1-yl)methyl)benzonitrile, Compound **X21**

Deamination was performed following the procedure described in refs (66) and (39).

Chemical formula: C_12_H_11_N_3_O.

White solid; melting point 167.3–168.5 °C. (Lit 168.0 °C);^1,2^ LC-MS *R*_t_ 0.51 min *m*/*z* = 214.20 MeCN; HRMS calculated for C_12_H_12_N_3_O as 214.0980, found 214.0980 (Δ = 0.0 ppm); ^1^H NMR (400 MHz, DMSO-*d*_6_) δ 7.86–7.81 (app. d, *J* = 8.3 Hz, 2H), 7.73 (d, *J* = 1.1 Hz, 1H), 7.34–7.28 (app. d, *J* = 8.3 Hz, 2H), 6.87 (s, 1H), 5.36 (s, 2H), 4.31 (s, 2H); ^13^C NMR (101 MHz, DMSO-*d*_6_) δ 143.91 (C), 139.17 (CH), 133.05 (2 × CH), 132.09 (C), 128.23 (2 × CH), 128.08 (CH), 119.14 (C), 110.75 (C), 53.14 (CH_2_), 47.58 (CH_2_); IR ν = 3128 br w, 2927 br w, 2846 br w, 2231 m, 1609 w, 1565 w, 1495 m, 1415 m, 1326 w, 1248 m, 1211 w, 1104 m, 1027 s, 966 w, 934 w cm^–1^.

#### 4-((5-((4-(3-Chlorophenyl)-3-oxopiperazin-1-yl)methyl)-1*H*-imidazol-1-yl)methyl)benzonitrile, Compound **X22**

The compound was prepared according to the procedure outlined in ref (39).

Orange solid; melting point 73.0 °C (decompose) (**X22**·H_2_O form lit. m.p. 90.0 °C).^39^ LC-MS *R*_t_ 1.43 min *m*/*z* = 406.35 MeCN; HRMS calculated for C_22_H_21_ClN_5_O as 406.1429, found 406.1432 (Δ = 0.7 ppm); ^13^C NMR (101 MHz, DMSO-*d*_6_) δ 166.04 (C), 143.67 (C), 143.60 (C), 140.68 (CH), 133.34 (C), 132.91 (CH), 130.81 (CH), 128.87 (CH), 128.25 (CH), 126.71 (CH), 126.15 (CH), 124.53 (CH), 119.00 (C), 111.50 (C), 56.95 (CH_2_), 50.46 (CH_2_), 49.16 (CH_2_), 48.75 (CH_2_), 48.59 (CH_2_); IR ν = 3394 br w, 3068 br w, 2229 w, 1657 s, 1592 m, 1480 m, 1419 m, 1341 s, 1323 m, 1160 m, 1114 m, 1080 w, 1021 w cm^–1^.

### Computational Methods

#### Computational Investigations Made with the LigAdvisor Web Server

The investigated compounds were each separately sketched into a dedicated “Structure search” input box available in the “Search in LigAdvisor” panel of the LigAdvisor web server (https://ligadvisor.unimore.it/, accessed on July 2nd, 2021).^26^ The searches were carried out by selecting the “MACCS or ECFP4” type of similarity and setting minimum thresholds of 80 and 30% (i.e., Tanimoto Indexes equal to 0.8 and 0.3) for MACCS and ECFP4 fingerprints, respectively. Similarity records and related target annotations of DrugBank and PDB ligands were than analyzed in KNIME.^67^

#### CYP19A1, CYP11B1, and CYP11B2 Data Set Generation

##### ChEMBL Data Set

CYP19A1, CYP11B1, and CYP11B2 inhibitors were collected from the ChEMBL database (accessed on May 1st, 2020) and filtered to retain molecules that have reported activity annotations complying with the following criteria:

- Target type equal to “Single Protein”;
- Standard type expressed as *K*_i_, *K*_d_, IC_50_, EC_50_, potency;
- Standard relation equal to “>” or “=”.

Moreover, filtered compounds were also desalted, and molecules with a molecular weight higher than 900 Da were removed. This phase of the ChEMBL data set preparation was performed by means of an in-house developed KNIME workflow.^67^ Afterward, the most relevant ionization and tautomeric states potentially accessible at a physiological pH by the prefiltered known inhibitors were generated with the *LigPrep* utility.^68^ Default settings were used in this phase of the preparation of CYP19A1, CYP11B1, and CYP11B2 inhibitors, except for the generation of every possible stereoisomer for compounds with undefined stereochemistry. Subsequently, up to 50 conformers were generated for each of the ligands with the *oeomega* module (OpenEye).^69^ A cutoff of 0.5 Å on root-mean-square deviation (RMSD) and an energy window of 10 kcal/mol were used as parameters to accept conformers during the conformational sampling.

##### DrugBank Data Set

The open data set of DrugBank compounds was first downloaded (accessed on May 1, 2023) and associated with target activity annotations. Only compounds with annotations on CYP19A1, CYP11B1, and CYP11B2 were retained. Again, ligands with a molecular weight higher than 900 Da were removed and desalted, by means of an in-house developed KNIME workflow. Afterward, the compounds were prepared, and their multiconformers were generated for the 3D similarity estimations using the same modalities described for the ChEMBL data set (vide supra).

##### PDB Data Set

X-ray crystallographic complexes of CYP19A1, CYP11B1, and CYP11B2 were first retrieved from the Protein Data Bank (accessed on May 1st, 2020).^43^ Then, their cocrystallized ligands were manually extracted in their bioactive conformation. Afterward, the compounds were filtered to retain only those accommodating in proximity to the HEME group and with a molecular weight ranging from 100 to 900 Da. Potential issues in the tautomerization state, atom typing, and in their stereochemistry were fixed, and hydrogen atoms were eventually added.

#### 3D Ligand-Based Analyses

The investigated compounds were first sketched with the 2DSketcher utility implemented in Maestro of the Schrodinger suite and then prepared for the 3D similarity estimations as follows. Two databases containing up to 5 and 50 conformers of each synthesized ligand were generated with default settings of the *oeomega* module (OpenEye),^69^ the first one being employed in the similarity assessments against the DrugBank and ChEMBL curated data sets, the second one being used in the ligand-based estimations against PDB cocrystallized compounds (vide supra). The similarity profile of each synthesized ligand was subsequently calculated with respect to compounds with activity annotations reported for CYP19A1, CYP11B1, and CYP11B2 into the DrugBank, ChEMBL and PDB databases, through a series of 3D similarity screenings made with ROCS (OpenEye).^53^ Default settings were used in all of the performed 3D similarity estimations, except for the selection of the queries for the screenings. In particular, the native poses of the CYP19A1, CYP11B1, and CYP11B2 crystallographic ligands were used as queries in the similarity assessments of the synthesized compounds against the curated PDB data set. Conversely, a multiconformers vs multiconformer approach was applied to evaluate the similarities with respect to compounds in the curated DrugBank and ChEMBL data sets. The *Tanimoto Combo* coefficient was selected as a metric to establish ligand similarity, with a threshold of 1.5 according to literature data.^52^ Postprocessing and statistics of the 3D similarity screenings were performed with KNIME.^67^

#### In Silico Tautomer Stability Assessment for Compound **X1**

The tautomeric preference of compound **X1** was evaluated by using the *Geometry Optimization* and *Single Point Energy* protocols available in Jaguar (Schrödinger).^62^ Default settings were used for the calculations, which were carried out with the DFT theory level, a B3LYP/3-61G** basis set, and an extended DFT grid and by selecting PBF-Water as solvent model.

#### Structure-Based Analyses

The structural complementarity of the synthesized compounds with the CYP19A1, CYP11B1, and CYP11B2 binding sites was also evaluated by means of molecular docking calculations performed with FRED (OpenEye).^58^ To this aim, the 3EQM,^59^ 4FDH,^51^ and 6M7X^49^ crystal structures were selected as representative conformations of the CYP19A1, CYP11B2, and CYP11B1 enzymes, respectively. In particular, the 3EQM PDB complex was selected as a representative structure of CYP19A1 due to the unavailability of complexes of this target with nonsteroidal ligands, and because of its higher resolution (i.e. 2.9 Å).^59^ 4FDH^51^ and 6M7X^49^ PDB complexes were selected as representatives of the CYP11B2 and CYP11B1 enzymes, respectively, as they have been cocrystallized with (*R*)-fadrozole (PDB ID: 4FDH; PDB ligand ID: 0T3) and (*S*)-fadrozole (PDB ID: 6M7X; PDB ligand ID: JTD), which emerged in the similarity estimations; the selection of these structures is in line with good practices of structure-based multitarget drug design.^70,71^ The selected structures were first prepared for docking by using default parameters via the Protein Preparation Wizard utility (Schrödinger).^72^ Receptor grids were generated by means of the *Make_receptor* application (OpenEye). Default parameters were used for the generation of the 3EQM, 4FDH, and 6M7X receptor grids, which were centered on the coordinates of their cocrystallized ligands. The HEME group present in the structures was considered to be part of the receptor during the generation of the grids and in the following docking process. Once the grids were generated, redocking calculations were performed in order to assess the ability of the docking protocol to reproduce the native binding mode (RMSDs between the redocking and crystallographic poses below 2.0 Å) (Figure S1e–g). Finally, the validated docking models were used to predict the binding mode of the synthesized compounds into the CYP19A1, CYP11B2, and CYP11B1 selected crystal structures. Docking scores were analyzed and compared to those obtained for the native ligands. The predicted docking poses were visually inspected, and the best candidates were finally selected.

### Biological Assays

#### In Vitro Assays on Recombinant CYP19A1, CYP1A2, and CYP3A4 Enzymes

The IC_50_ value of compound **X21** against CYP19A1 was evaluated by means of an Aromatase (CYP19A1) Inhibitor Screening Kit from BioVision. The testes compound was first suspended at a concentration of 10 mM, and then tested in 10-dose IC_50_ mode with a 3-fold serial dilution, starting from a concentration of 10 μM. Letrozole was used as a control in this assay (starting from a 1 μM concentration). The in vitro tests on Aromatase were performed by using the Kit Cat# K984-100 assay kit; the Regeneration System, 100× and NADP^+^: (100×), 10 mM were used as reaction buffer. In particular, the enzyme was first prepared with the Regeneration System and substrate with NADP^+^ in freshly prepared reaction buffer. Then, the resulting solution was delivered into the reaction well. Afterward, compound **X21** and the control compound were delivered into the enzyme solution by Acoustic technology (Echo550; nanoliter range) and incubated for 20 min at room temperature. Subsequently, a solution of the CYP19A1 substrate was delivered into the reaction well to initiate the reaction at 37 °C. The enzyme activities were monitored as a time-course measurement of the increase in fluorescence signal from fluorescence substrate for 60 min, at 37 °C in EnVision (Ex 485/Em 535 nm).

The assays on CYP1A2 and CYP3A4 were based on the fluorescence read out using Vivid fluorescence substrates against CYP BACULOSOMES from ThermoFisher Scientific. The test compound was first suspended at a concentration of 10 mM and tested in 10-dose IC_50_ mode with a 3-fold serial dilution, starting from a concentration of 10 μM. The in vitro tests on CYP1A2 and CYP3A4 were performed by using the Kit Cat# P2863 and P2858 assay kits, respectively; 100 mM potassium phosphate buffer (pH 8.0), and 1% DMSO, Vivid Regeneration System, 100× (333 mM glucose-6-phosphate and 30 U/mL glucose-6-phosphate dehydrogenase in 100 mM potassium phosphate, pH 8.0) and NADP^+^: (100×), 10 mM were used as reaction buffer. IC_50_ values of compound **X21** against CYP3A4 and CYP1A2 were determined with the same modalities described above, except for: (i) the use of control compounds Ketoconazole (CYP3A4), and Furafylline (CYP1A2), which were tested in a 10-dose IC_50_ mode with 3-fold serial dilution starting from 1 and 20 μM, respectively; (ii) the use of substrates specific for CYP1A2 (10 μM Vivid EOMCC Substrate) and CYP3A4 (10 μM Vivid BOMCC Substrate), and; (iii) enzyme activity monitoring, which was determined through a time-course measurement of the increase in fluorescence signal from fluorescence substrate for 100 min at room temperature in EnVision (Ex 405/Em 460 nm).

### Pharmacological Assays

#### Materials, Drugs, and Cells Lines

Recombinant human epidermal growth factor (EGF) and basic fibroblast growth factor (bFGF) were obtained from PeproTechEC LTD (London, UK). Cell culture media, MCDB-131, RPMI-1640, fetal bovine serum (FBS), l-glutamine, and antibiotics were from Gibco (ThermoFisher Scientific, Waltham, MA, USA). Type A gelatin from porcine skin, supplements, and all other chemicals not listed in this section were from Sigma Chemical Co. (St. Louis, MO, USA). Plastics for cell culture were supplied by Sarstedt (Nümbrecht, Germany).

The human breast cancer cell lines MCF-7 and MDA-MB-231 were obtained from the American Type Culture Collection (ATCC; Manassas, USA) and maintained in 20% FBS RPMI-1640 medium supplemented with antibiotics and 2 mM l-glutamine, whereas human normal dermal human fibroblast cells (HNDF; ATCC) were maintained in MCDB-131 culture medium supplemented with antibiotics, 20% heat-inactivated FBS, l-glutamine (2 mM), heparin (10 IU/mL), rhEGF (10 ng/mL), and rhbFGF (5 ng/mL). Cell lines were routinely grown in tissue culture flasks, covered with type A gelatin only for HNDF, and kept in a humidified atmosphere of 5% CO_2_ at 37 °C.

In vitro pharmacological studies were performed using drugs diluted from a 10 mM stock solution (in 100% dimethyl sulfoxide). DMSO concentration in the control’s media was the one utilized to dilute the highest concentration of compound **X21** in the medium of treated samples for the same experiment.

#### Cell Proliferation and Apoptosis Assay

MCF-7 and MDA-MB-231 cells were plated in 24-well plates and allowed to attach overnight. Cells were treated with compound **X21** (0.001–50 μM) or with its vehicle for 24, 48, and 72 h. HNDF cells were exposed to compound **X21** for 72 h, whereas MCF-7 cells were also treated for 72 h with letrozole (0.001–50 μM), as a positive control. At the end of the treatment, viable cells (evaluated by trypan blue dye exclusion) were counted with a hemocytometer. The concentration of drug that reduced cell proliferation by 50% (IC_50_) vs controls was calculated by nonlinear regression fit of the mean values of data obtained in triplicate experiments (at least nine wells for each concentration).

To quantify apoptosis induced by compound **X21**, 30 × 10^4^ MCF-7 or MDA-MB-231 cells were plated in 100 mm sterile dishes and treated for 24 h with different concentrations of **X21** (0.35, 0.7, and 1 μM for MCF-7; 10, 40, and 50 μM for MDA-MB-231, and with vehicle alone. At the end of the incubation, cells were collected, and the samples were analyzed with the cell death detection enzyme-linked immunosorbent assay (ELISA) Plus kit (Roche, Switzerland). All experiments were repeated three times with at least three replicates per sample.

#### Luminex Analysis

MCF-7 cells (5 × 10^4^) were plated and treated with compound **X21** (700 nM, the experimental antiproliferative IC_50_) and vehicle alone for 24 h (three replicates per sample). At the end of the experiment, the cells were lysed at 4 °C with Milliplex lysis buffer supplemented with protease inhibitors, and then the samples were filtered with Ultrafree-MC centrifugal filter devices with microporous membranes from MerckMillipore (Merck KGaA, Darmstadt, Germany). Twenty-five microliters of filtered lysate was diluted in assay buffer (1:2 v:v, respectively), and then a 25 μL sample of the solution was evaluated by Luminex using the MILLIPLEX Akt/mTOR Phosphoprotein 11-plex Magnetic Bead kit (catalogue #48-611MAG kit) purchased from MerckMillipore. The samples were loaded into a 96-well plate supplied by the kit. In each well, an equal volume of a premix of 11 luminex beads was added, followed by incubation overnight at 4 °C. The beads were subsequently washed and incubated with 25 μL of secondary biotinylated detection antibody for 1 h at room temperature, according to the manufacturer’s protocol. The samples were analyzed by a FlexMap3D instrument (MerckMillipore) with xPONENT software (MerckMillipore) following the manufacturer’s protocols and settings. Results were reported as the percentage of the phosphorylated protein/total protein ratio vs 100% of vehicle-treated cells.

#### Statistical Data Analysis

The analysis by ANOVA, followed by the Student–Newman–Keuls test, was used to assess the statistical differences of pharmacological data in vitro. *P*-values lower than 0.05 were considered significant. Statistical analyses were performed using the GraphPad Prism software package, version 5.0 (GraphPad Software Inc., San Diego, CA, USA).

#### Cardiac Safety Assessment

Compound activity against the voltage-gated potassium channel *h*ERG and sodium channel Nav1.5 was assessed to evaluate the potential cardiac liabilities. The assays on *h*ERG were performed by means of the Manual *h*ERG Patch Clamp Assay in CHO-*h*ERG cells. To this aim, electrodes (2.5–4 MW) were filled with intracellular solution (in mM): KCl (120), HEPES (10), CaCl_2_ (10), MgCl_2_ (1.7), EGTA (10), K_2_ATP (4), pH 7.2, approximately 290 mOsM. Cells were continuously perfused in extracellular solution containing (in mM): NaCl (145), KCl (4) CaCl_2_ (2), MgCl_2_ (1), HEPES (10), pH 7.4, approximately 305 mOsM. The voltage protocol in this assay started with a holding potential equal to −80 mV and hyperpolarization equal to +40 mV for 500 ms, followed by a ramp of 100 ms with −80 mV potential, repeated every 5 s. *h*ERG current is defined as peak current elicited by Ramp in pA. Compound **X21** was added via continuous perfusion until the *h*ERG current reached a plateau. Six concentrations of compound **X21** were added from lowest concentration to highest (10 μM), with a 3-factor dilution. A 10-μM solution of E-4031 was added after the **X21** curve was completed to establish full blockade of the *h*ERG current. All recordings were performed at room temperature.

The assays on sodium Nav1.5 ion channel were performed by means of Manual Patch Clamp Assay, in HEK-NaV1.5 stable cells. To this aim, GC150TF-10 electrodes (1.5 Outer diameter × 1.17 inner diameter × 100 length [mm], 3–5 MOhm) were filled with intracellular solution (in mM): KCl (120), HEPES (10), CaCl_2_ (5), MgCl_2_ (1.7), K_2_ATP (4), EGTA (10), pH 7.2, approximately 290 mOsM. Cells were continuously perfused in extracellular solution containing (in mM): NaCl (145), KCl (4), CaCl_2_ (2), MgCl_2_ (1), HEPES (10), d-glucose (10), pH 7.4, approximately 305 mOsM.

The voltage protocol was conducted first with a holding potential equal to −80 mV and hyperpolarization equal to −120 mV for 500 ms, followed by a step of 4 ms with a −15 mV potential, repeated every 5 s. Nav1.5 current was defined as negative peak current elicited by Step to −15 mV in pA. Compound **X21** was tested in 6-point IC_50_ mode, with a 3-fold dilution, starting at a maximum concentration of 30 μM. DMSO was added to all compound solutions up to a final concentration of 0.3%. The control compound tetrodotoxin was tested in 7-point IC_50_ mode, with a 3-fold dilution starting at a maximum concentration of 10 μM. All recordings were performed at room temperature.

## Supporting Information Available

Results of the similarity estimations performed with the LigAdvisor Web server; results of the similarity estimations performed with respect to compounds with activity annotation on CYP19A1, CYP11B2, and CYP11B1, reported in the DrugBank, PDB, and ChEMBL databases; docking scores of the investigated compounds into the PDB crystal structures 3EQM (CYP19A1), 6M7X (CYP11B1), and 4FDH (CYP11B2); binding mode predicted for (*S*)- and (*R*)-fadrozole into the CYP19A1 binding site (PDB ID: 3EQM); titration curves of compound X21 against CYP19A1, CYP1A2, and CYP3A4, with their respective controls; antiproliferative in vitro activity of fadrozole on human MCF-7 (Estrogen Receptor+) at 24, 48, and 72 h; and titration curves of compound **X21** and the reference compounds E-4031 and tetrodoxin, against *h*ERG and Nav1.5 (manual patch clamp assays), respectively (PDF)

## Abbreviations

ACE: angiotensin-converting enzyme
AI: aromatase inhibitor
AKT: protein kinase B
Ang II: angiotensin II
AT1R: angiotensin type 1 receptor
ATCC: American Type Culture Collection
BC: breast cancer
bFGF: basic fibroblast growth factor
C: vehicle-treated control
CVD: cardiovascular disease
CYP11B1: cytochrome P450 family 11 subfamily B member 1
CYP11B2: aldosterone synthase
CYP19A1: aromatase
CYP1A2: cytochromes P450 1A2
CYP3A4: cytochromes P450 3A4
EGF: epidermal growth factor
ERBB2/HER2+: human epidermal growth factor receptor-2 positive
ER: estrogen receptor
FBS: foetal bovine serum
GSK: glycogen synthase kinase 3
HNDF: human normal dermal human fibroblast cell line
IGF1R: insulin-like growth factor 1 (IGF-1) receptor
IR: insulin receptor
IRS1: insulin receptor substrate 1
mTOR: mammalian target of rapamycin
MW: molecular weight
p70S6K: ribosomal protein S6 kinase beta-1
PDB: protein data bank
PR: progesterone receptor
PTEN: phosphatase and tensin homologue
RAAS: renin–angiotensin–aldosterone system
RMSD: root-mean-square deviation
RP6S: ribosomal protein S6
SERD: selective estrogen receptors degrader
SERM: selective estrogen receptors modulator
TSC2: tuberous sclerosis complex 2
