# Supplementary material for: Discovery of a Potent Dual Inhibitor of Aromatase and Aldosterone Synthase
Source: ACS Pharmacol Transl Sci. 2023 Nov 23;6(12):1870–83. doi: 10.1021/acsptsci.3c00183 (PMC10714424; doi:10.1021/acsptsci.3c00183)

SUPPORTING INFORMATION

Discovery of a potent dual inhibitor of aromatase and aldosterone synthase

Annachiara Tinivella,[a] Marta Banchi,[b] Guido Gambacorta,[c] Federica Borghi,[a] Paola Orlandi,[b] Ian R. Baxendale,[c] Antonello Di Paolo,[b] Guido Bocci,[b] Luca Pinzi,[a]* Giulio Rastelli[a]*

[a] *Department of Life Sciences, University of Modena and Reggio Emilia, Via G. Campi 103 – 41125 Modena, Italy.*

[b] *Department of Clinical and Experimental Medicine, University of Pisa, Via Roma 55, 56126 Pisa, Italy*

[c] *Department of Chemistry, University of Durham, Durham, UK.*

Corresponding Authors:

For Editorial correspondence:
Prof. Giulio Rastelli
Department of Life Sciences
University of Modena and Reggio Emilia
Via G. Campi 103 – 41125 Modena, Italy.
Phone: +39 059 2058564
E-mail: giulio.rastelli@unimore.it

Dr. Luca Pinzi
Department of Life Sciences
University of Modena and Reggio Emilia
Via G. Campi 103 – 41125 Modena, Italy.
Phone: +39 059 2058625
E-mail: luca.pinzi@unimore.it

Table of Contents

Table S1…………………………………………………………………………………………….. 3
Table S2…………………………………………………………………………………………….. 6
Table S3……….……………………………………………………………………………………... 11

Figure S1……………………………………………………………………………………..…… 12
Figure S2…………………………………………………………………………………………...13
Figure S3……………………………………………………………………………….……….…14
Figure S4……………………………………………………………………………….……….…15

Table S1: Results of the similarity estimations performed with the LigAdvisor webserver. For each synthesized compound, the DrugBank and PDB ligands with similarity values above thresholds are reported. Only similarity records above thresholds (ECFP4*fp* Tanimoto index ≥ 0.3 and *TanimotoCombo* score ≥ 1.5) are reported.

Synthetised compound	*DrugBank ligand*	*MACCSfp Tanimoto Index*	*ECFP4fp Tanimoto Index*	*Protein Name*	*PDB IDs*
X24	JD7	0.435	0.31	Cytochrome P450 11B1, mitochondrial	[6M7X]
X20	JD7	0.476	0.321	Cytochrome P450 11B1, mitochondrial	[6M7X]
X22	JD7	0.49	0.341	Cytochrome P450 11B1, mitochondrial	[6M7X]
X21	JD7	0.5	0.481	Cytochrome P450 11B1, mitochondrial	[7M8V]
X21	YSY	0.524	0.4	Cytochrome P450 11B2, mitochondrial	[6XZ9]
X2	O4W	0.547	0.31	Cytochrome P450 11B2, mitochondrial	[6XZ9]
X6	O4W	0.508	0.31	Cytochrome P450 11B2, mitochondrial	[4FDH, 7M8I]
X24	0T3	0.435	0.31	Cytochrome P450 11B2, mitochondrial	[4FDH, 7M8I]
X24	0T3	0.435	0.31	Cytochrome P450 11B2, mitochondrial	[6XZ8]
X22	O4T	0.507	0.311	Cytochrome P450 11B2, mitochondrial	[7M8V]
X1	YSY	0.442	0.312	Cytochrome P450 11B2, mitochondrial	[7M8V]
X2	YSY	0.478	0.314	Cytochrome P450 11B2, mitochondrial	[7M8V]
X6	YSY	0.523	0.314	Cytochrome P450 11B2, mitochondrial	[6XZ8]
X8	O4T	0.389	0.316	Cytochrome P450 11B2, mitochondrial	[6XZ9]
X3	O4W	0.565	0.317	Cytochrome P450 11B2, mitochondrial	[4FDH, 7M8I]
X20	0T3	0.476	0.321	Cytochrome P450 11B2, mitochondrial	[4FDH, 7M8I]
X20	0T3	0.476	0.321	Cytochrome P450 11B2, mitochondrial	[7M8V]
X3	YSY	0.5	0.324	Cytochrome P450 11B2, mitochondrial	[6XZ8]
X13	O4T	0.419	0.324	Cytochrome P450 11B2, mitochondrial	[6XZ8]
X2	O4T	0.403	0.333	Cytochrome P450 11B2, mitochondrial	[6XZ8]
X6	O4T	0.37	0.333	Cytochrome P450 11B2, mitochondrial	[6XZ9]
X13	O4W	0.463	0.333	Cytochrome P450 11B2, mitochondrial	[4FDH, 7M8I]
X22	0T3	0.49	0.341	Cytochrome P450 11B2, mitochondrial	[4FDH, 7M8I]
X22	0T3	0.49	0.341	Cytochrome P450 11B2, mitochondrial	[6XZ8]
X3	O4T	0.435	0.343	Cytochrome P450 11B2, mitochondrial	[6XZ9]
X22	O4W	0.662	0.347	Cytochrome P450 11B2, mitochondrial	[7M8V]
X22	YSY	0.481	0.357	Cytochrome P450 11B2, mitochondrial	[4FDH, 7M8I]
X21	0T3	0.5	0.481	Cytochrome P450 11B2, mitochondrial	[4FDH, 7M8I]

Synthetised compound	*DrugBank ligand*	*MACCSfp Tanimoto Index*	*ECFP4fp Tanimoto Index*	*Protein Name*
X1	DB01217	0.4	0.333	Aromatase
X1	DB03467	0.172	0.333	Aromatase
X2	DB00357	0.323	0.312	Aromatase
X2	DB01217	0.429	0.333	Aromatase
X2	DB03467	0.214	0.419	Aromatase
X3	DB00357	0.311	0.323	Aromatase
X3	DB01217	0.447	0.345	Aromatase
X3	DB03467	0.222	0.433	Aromatase

X4	DB03467	0.2	0.323	Aromatase
X6	DB00357	0.306	0.312	Aromatase
X6	DB01217	0.438	0.333	Aromatase
X6	DB03467	0.218	0.419	Aromatase
X7	DB01217	0.429	0.5	Aromatase
X8	DB00655	0.226	0.317	Aromatase
X8	DB01217	0.438	0.312	Aromatase
X8	DB03467	0.218	0.394	Aromatase
X9	DB01217	0.407	0.355	Aromatase
X10	DB01217	0.407	0.407	Aromatase
X11	DB01217	0.338	0.4	Aromatase
X12	DB01217	0.338	0.4	Aromatase
X13	DB01217	0.338	0.323	Aromatase
X13	DB03467	0.187	0.324	Aromatase
X14	DB01217	0.333	0.312	Aromatase
X14	DB03467	0.169	0.314	Aromatase
X15	DB01217	0.393	0.357	Aromatase
X16	DB01217	0.412	0.31	Aromatase
X17	DB01217	0.303	0.364	Aromatase
X18	DB01217	0.431	0.391	Aromatase
X19	DB01217	0.431	0.36	Aromatase
X20	DB01217	0.512	0.435	Aromatase
X21	DB01006	0.475	0.478	Aromatase
X21	DB01217	0.535	0.44	Aromatase
X22	DB00655	0.21	0.312	Aromatase
X22	DB03467	0.222	0.31	Aromatase
X23	DB00184	0.432	0.321	Aromatase
X23	DB01217	0.548	0.357	Aromatase
X24	DB01217	0.468	0.417	Aromatase
X1	DB00648	0.208	0.346	Cytochrome P450 11B1, mitochondrial
X1	DB11837	0.442	0.312	Cytochrome P450 11B1, mitochondrial
X2	DB00648	0.244	0.3	Cytochrome P450 11B1, mitochondrial
X2	DB05667	0.603	0.318	Cytochrome P450 11B1, mitochondrial
X2	DB11837	0.478	0.314	Cytochrome P450 11B1, mitochondrial
X3	DB00648	0.256	0.31	Cytochrome P450 11B1, mitochondrial
X3	DB05667	0.571	0.326	Cytochrome P450 11B1, mitochondrial
X3	DB11837	0.5	0.324	Cytochrome P450 11B1, mitochondrial
X4	DB00648	0.196	0.333	Cytochrome P450 11B1, mitochondrial
X5	DB00648	0.163	0.36	Cytochrome P450 11B1, mitochondrial
X6	DB00648	0.19	0.3	Cytochrome P450 11B1, mitochondrial
X6	DB05667	0.562	0.318	Cytochrome P450 11B1, mitochondrial
X6	DB11837	0.523	0.314	Cytochrome P450 11B1, mitochondrial
X7	DB01011	0.289	0.333	Cytochrome P450 11B1, mitochondrial
X8	DB00648	0.19	0.323	Cytochrome P450 11B1, mitochondrial
X8	DB05667	0.562	0.304	Cytochrome P450 11B1, mitochondrial
X11	DB01011	0.197	0.393	Cytochrome P450 11B1, mitochondrial

X12	DB01011	0.197	0.393	Cytochrome P450 11B1, mitochondrial
X13	DB01011	0.197	0.31	Cytochrome P450 11B1, mitochondrial
X14	DB05667	0.5	0.304	Cytochrome P450 11B1, mitochondrial
X15	DB00648	0.229	0.321	Cytochrome P450 11B1, mitochondrial
X17	DB01011	0.176	0.312	Cytochrome P450 11B1, mitochondrial
X18	DB01011	0.22	0.318	Cytochrome P450 11B1, mitochondrial
X19	DB00648	0.125	0.32	Cytochrome P450 11B1, mitochondrial
X20	DB01011	0.262	0.304	Cytochrome P450 11B1, mitochondrial
X21	DB11837	0.524	0.4	Cytochrome P450 11B1, mitochondrial
X22	DB05667	0.746	0.38	Cytochrome P450 11B1, mitochondrial
X22	DB11837	0.481	0.357	Cytochrome P450 11B1, mitochondrial
X23	DB01011	0.395	0.346	Cytochrome P450 11B1, mitochondrial
X24	DB00648	0.22	0.32	Cytochrome P450 11B1, mitochondrial
X1	DB11837	0.442	0.312	Cytochrome P450 11B2, mitochondrial
X2	DB05667	0.603	0.318	Cytochrome P450 11B2, mitochondrial
X2	DB11837	0.478	0.314	Cytochrome P450 11B2, mitochondrial
X3	DB05667	0.571	0.326	Cytochrome P450 11B2, mitochondrial
X3	DB11837	0.5	0.324	Cytochrome P450 11B2, mitochondrial
X6	DB05667	0.562	0.318	Cytochrome P450 11B2, mitochondrial
X6	DB11837	0.523	0.314	Cytochrome P450 11B2, mitochondrial
X8	DB05667	0.562	0.304	Cytochrome P450 11B2, mitochondrial
X14	DB05667	0.5	0.304	Cytochrome P450 11B2, mitochondrial
X21	DB11837	0.524	0.4	Cytochrome P450 11B2, mitochondrial
X22	DB05667	0.746	0.38	Cytochrome P450 11B2, mitochondrial
X22	DB11837	0.481	0.357	Cytochrome P450 11B2, mitochondrial

Table S2: Results of the similarity estimations performed with respect to compounds with activity annotation on CYP19A1, CYP11B2 and CYP11B1, reported in the DrugBank, PDB and ChEMBL databases. For each synthesized compound, the DrugBank, PDB and ChEMBL ligands with similarity values above thresholds are reported (*TanimotoCombo* score ≥ 1.5).

Synthetised compound	*PDB ligand ID*	*Tanimoto Combo*	*Shape Tanimoto*	*Color Tanimoto*	*Protein Name*	*PDB IDs*
X21	JD7	1,518	0,9	0,618	Cytochrome P450 11B1, mitochondrial	[6M7X]
X21	0T3	1,555	0,93	0,625	Cytochrome P450 11B2, mitochondrial	[4FDH]

Synthesized compound	*DrugBank ligand*	*Tanimoto Combo*	*Shape Tanimoto*	*Color Tanimoto*	*Protein Name*
X21	DB11837	1,542	0,918	0,624	Cytochrome P450 11B1, mitochondrial, mitochondrial

Synthesized compound	*ChEMBL ligand*	*Tanimoto Combo*	*Shape Tanimoto*	*Color Tanimoto*	*Protein Name*
X1	CHEMBL599601	1,675	0,926	0,75	Cytochrome P450 11B1, mitochondrial
X1	CHEMBL681	1,513	0,802	0,711	Cytochrome P450 11B1, mitochondrial
X1	CHEMBL23731	1,513	0,802	0,711	Cytochrome P450 11B1, mitochondrial
X1	CHEMBL611680	1,512	0,906	0,606	Cytochrome P450 11B1, mitochondrial
X2	CHEMBL681	1,912	0,917	0,996	Cytochrome P450 11B1, mitochondrial
X2	CHEMBL23731	1,912	0,917	0,996	Cytochrome P450 11B1, mitochondrial
X2	CHEMBL611680	1,677	0,844	0,833	Cytochrome P450 11B1, mitochondrial
X2	CHEMBL599622	1,58	0,715	0,865	Cytochrome P450 11B1, mitochondrial
X2	CHEMBL599623	1,578	0,713	0,865	Cytochrome P450 11B1, mitochondrial
X2	CHEMBL608140	1,574	0,708	0,865	Cytochrome P450 11B1, mitochondrial
X2	CHEMBL1649605	1,559	0,759	0,8	Cytochrome P450 11B1, mitochondrial
X2	CHEMBL599222	1,551	0,885	0,666	Cytochrome P450 11B1, mitochondrial
X2	CHEMBL1651492	1,545	0,745	0,8	Cytochrome P450 11B1, mitochondrial
X2	CHEMBL1651495	1,531	0,731	0,8	Cytochrome P450 11B1, mitochondrial
X2	CHEMBL599601	1,522	0,832	0,691	Cytochrome P450 11B1, mitochondrial
X2	CHEMBL598390	1,51	0,71	0,8	Cytochrome P450 11B1, mitochondrial
X2	CHEMBL1651493	1,508	0,709	0,8	Cytochrome P450 11B1, mitochondrial
X2	CHEMBL608437	1,507	0,707	0,8	Cytochrome P450 11B1, mitochondrial
X3	CHEMBL681	1,89	0,895	0,996	Cytochrome P450 11B1, mitochondrial
X3	CHEMBL23731	1,89	0,895	0,996	Cytochrome P450 11B1, mitochondrial
X3	CHEMBL611680	1,72	0,887	0,833	Cytochrome P450 11B1, mitochondrial
X3	CHEMBL1649605	1,594	0,795	0,8	Cytochrome P450 11B1, mitochondrial
X3	CHEMBL1651492	1,583	0,784	0,8	Cytochrome P450 11B1, mitochondrial
X3	CHEMBL1651495	1,562	0,763	0,8	Cytochrome P450 11B1, mitochondrial
X3	CHEMBL599601	1,561	0,871	0,691	Cytochrome P450 11B1, mitochondrial
X3	CHEMBL598390	1,545	0,745	0,8	Cytochrome P450 11B1, mitochondrial
X3	CHEMBL1651493	1,544	0,744	0,8	Cytochrome P450 11B1, mitochondrial
X3	CHEMBL608437	1,542	0,742	0,8	Cytochrome P450 11B1, mitochondrial
X3	CHEMBL212741	1,532	0,734	0,798	Cytochrome P450 11B1, mitochondrial
X3	CHEMBL377770	1,532	0,733	0,798	Cytochrome P450 11B1, mitochondrial
X3	CHEMBL1651494	1,53	0,731	0,8	Cytochrome P450 11B1, mitochondrial
X3	CHEMBL1328035	1,52	0,854	0,666	Cytochrome P450 11B1, mitochondrial
X3	CHEMBL162496	1,517	0,851	0,666	Cytochrome P450 11B1, mitochondrial
X3	CHEMBL1428763	1,517	0,851	0,666	Cytochrome P450 11B1, mitochondrial
X3	CHEMBL1444873	1,517	0,851	0,666	Cytochrome P450 11B1, mitochondrial
X3	CHEMBL599222	1,515	0,849	0,666	Cytochrome P450 11B1, mitochondrial
X3	CHEMBL599623	1,505	0,839	0,666	Cytochrome P450 11B1, mitochondrial
X3	CHEMBL599622	1,504	0,838	0,666	Cytochrome P450 11B1, mitochondrial
X3	CHEMBL610501	1,5	0,835	0,666	Cytochrome P450 11B1, mitochondrial
X5	CHEMBL599601	1,668	0,919	0,749	Cytochrome P450 11B1, mitochondrial
X5	CHEMBL681	1,514	0,803	0,711	Cytochrome P450 11B1, mitochondrial
X5	CHEMBL23731	1,514	0,803	0,711	Cytochrome P450 11B1, mitochondrial
X5	CHEMBL611680	1,51	0,903	0,607	Cytochrome P450 11B1, mitochondrial
X6	CHEMBL681	1,893	0,913	0,98	Cytochrome P450 11B1, mitochondrial
X6	CHEMBL23731	1,893	0,913	0,98	Cytochrome P450 11B1, mitochondrial
X6	CHEMBL611680	1,673	0,84	0,833	Cytochrome P450 11B1, mitochondrial

X6	CHEMBL599222	1,554	0,888	0,666	Cytochrome P450 11B1, mitochondrial
X6	CHEMBL599622	1,552	0,885	0,667	Cytochrome P450 11B1, mitochondrial
X6	CHEMBL599623	1,55	0,884	0,667	Cytochrome P450 11B1, mitochondrial
X6	CHEMBL1651492	1,549	0,75	0,8	Cytochrome P450 11B1, mitochondrial
X6	CHEMBL608140	1,529	0,862	0,667	Cytochrome P450 11B1, mitochondrial
X6	CHEMBL610501	1,528	0,864	0,664	Cytochrome P450 11B1, mitochondrial
X6	CHEMBL598390	1,515	0,715	0,8	Cytochrome P450 11B1, mitochondrial
X6	CHEMBL1651493	1,514	0,714	0,8	Cytochrome P450 11B1, mitochondrial
X6	CHEMBL591465	1,513	0,941	0,571	Cytochrome P450 11B1, mitochondrial
X6	CHEMBL608437	1,512	0,712	0,8	Cytochrome P450 11B1, mitochondrial
X6	CHEMBL598606	1,51	0,939	0,571	Cytochrome P450 11B1, mitochondrial
X6	CHEMBL463582	1,507	0,842	0,664	Cytochrome P450 11B1, mitochondrial
X6	CHEMBL1651494	1,503	0,703	0,8	Cytochrome P450 11B1, mitochondrial
X7	CHEMBL681	1,682	0,702	0,981	Cytochrome P450 11B1, mitochondrial
X7	CHEMBL23731	1,682	0,702	0,981	Cytochrome P450 11B1, mitochondrial
X8	CHEMBL681	1,832	0,853	0,978	Cytochrome P450 11B1, mitochondrial
X8	CHEMBL23731	1,832	0,853	0,978	Cytochrome P450 11B1, mitochondrial
X8	CHEMBL599622	1,548	0,881	0,667	Cytochrome P450 11B1, mitochondrial
X8	CHEMBL599623	1,546	0,879	0,667	Cytochrome P450 11B1, mitochondrial
X8	CHEMBL599222	1,544	0,878	0,666	Cytochrome P450 11B1, mitochondrial
X8	CHEMBL608140	1,526	0,859	0,667	Cytochrome P450 11B1, mitochondrial
X10	CHEMBL599601	1,533	0,784	0,75	Cytochrome P450 11B1, mitochondrial
X18	CHEMBL599601	1,6	0,851	0,749	Cytochrome P450 11B1, mitochondrial
X18	CHEMBL681	1,512	0,801	0,711	Cytochrome P450 11B1, mitochondrial
X18	CHEMBL23731	1,512	0,801	0,711	Cytochrome P450 11B1, mitochondrial
X19	CHEMBL599601	1,637	0,887	0,749	Cytochrome P450 11B1, mitochondrial
X19	CHEMBL681	1,541	0,831	0,711	Cytochrome P450 11B1, mitochondrial
X19	CHEMBL23731	1,541	0,831	0,711	Cytochrome P450 11B1, mitochondrial
X20	CHEMBL599601	1,766	0,909	0,857	Cytochrome P450 11B1, mitochondrial
X20	CHEMBL681	1,681	0,851	0,83	Cytochrome P450 11B1, mitochondrial
X20	CHEMBL23731	1,681	0,851	0,83	Cytochrome P450 11B1, mitochondrial
X20	CHEMBL611680	1,585	0,896	0,688	Cytochrome P450 11B1, mitochondrial
X20	CHEMBL610794	1,564	0,857	0,707	Cytochrome P450 11B1, mitochondrial
X20	CHEMBL1651492	1,549	0,882	0,667	Cytochrome P450 11B1, mitochondrial
X20	CHEMBL591465	1,549	0,843	0,707	Cytochrome P450 11B1, mitochondrial
X20	CHEMBL598606	1,546	0,839	0,707	Cytochrome P450 11B1, mitochondrial
X20	CHEMBL598390	1,545	0,879	0,666	Cytochrome P450 11B1, mitochondrial
X20	CHEMBL1651493	1,545	0,878	0,666	Cytochrome P450 11B1, mitochondrial
X20	CHEMBL377770	1,544	0,877	0,666	Cytochrome P450 11B1, mitochondrial
X20	CHEMBL608437	1,544	0,877	0,666	Cytochrome P450 11B1, mitochondrial
X20	CHEMBL212741	1,543	0,877	0,667	Cytochrome P450 11B1, mitochondrial
X20	CHEMBL608140	1,534	0,768	0,767	Cytochrome P450 11B1, mitochondrial
X20	CHEMBL1651494	1,52	0,854	0,667	Cytochrome P450 11B1, mitochondrial
X20	CHEMBL599622	1,52	0,753	0,767	Cytochrome P450 11B1, mitochondrial
X20	CHEMBL599623	1,518	0,752	0,767	Cytochrome P450 11B1, mitochondrial
X20	CHEMBL1649605	1,503	0,837	0,667	Cytochrome P450 11B1, mitochondrial
X21	CHEMBL599601	2	1	1	Cytochrome P450 11B1, mitochondrial
X21	CHEMBL611680	1,782	0,982	0,8	Cytochrome P450 11B1, mitochondrial
X21	CHEMBL162496	1,681	0,967	0,714	Cytochrome P450 11B1, mitochondrial
X21	CHEMBL349822	1,629	0,914	0,714	Cytochrome P450 11B1, mitochondrial
X21	CHEMBL599223	1,593	0,968	0,625	Cytochrome P450 11B1, mitochondrial
X21	CHEMBL9298	1,554	0,931	0,623	Cytochrome P450 11B1, mitochondrial
X21	CHEMBL31215	1,554	0,931	0,623	Cytochrome P450 11B1, mitochondrial
X21	CHEMBL599222	1,552	0,839	0,714	Cytochrome P450 11B1, mitochondrial
X21	CHEMBL598399	1,547	0,868	0,679	Cytochrome P450 11B1, mitochondrial
X21	CHEMBL3099695	1,542	0,918	0,624	Cytochrome P450 11B1, mitochondrial
X21	CHEMBL3099683	1,511	0,888	0,622	Cytochrome P450 11B1, mitochondrial
X21	CHEMBL681	1,502	0,791	0,712	Cytochrome P450 11B1, mitochondrial
X21	CHEMBL23731	1,502	0,791	0,712	Cytochrome P450 11B1, mitochondrial
X23	CHEMBL681	1,74	0,922	0,817	Cytochrome P450 11B1, mitochondrial
X23	CHEMBL23731	1,74	0,922	0,817	Cytochrome P450 11B1, mitochondrial
X24	CHEMBL599601	1,728	0,978	0,75	Cytochrome P450 11B1, mitochondrial
X24	CHEMBL211207	1,621	0,907	0,714	Cytochrome P450 11B1, mitochondrial

X24	CHEMBL611680	1,558	0,955	0,603	Cytochrome P450 11B1, mitochondrial
X24	CHEMBL681	1,542	0,83	0,712	Cytochrome P450 11B1, mitochondrial
X24	CHEMBL23731	1,542	0,83	0,712	Cytochrome P450 11B1, mitochondrial
X1	CHEMBL599601	1,675	0,926	0,75	Cytochrome P450 11B2, mitochondrial
X1	CHEMBL681	1,513	0,802	0,711	Cytochrome P450 11B2, mitochondrial
X1	CHEMBL23731	1,513	0,802	0,711	Cytochrome P450 11B2, mitochondrial
X1	CHEMBL611680	1,512	0,906	0,606	Cytochrome P450 11B2, mitochondrial
X2	CHEMBL681	1,912	0,917	0,996	Cytochrome P450 11B2, mitochondrial
X2	CHEMBL23731	1,912	0,917	0,996	Cytochrome P450 11B2, mitochondrial
X2	CHEMBL611680	1,677	0,844	0,833	Cytochrome P450 11B2, mitochondrial
X2	CHEMBL599622	1,58	0,715	0,865	Cytochrome P450 11B2, mitochondrial
X2	CHEMBL599623	1,578	0,713	0,865	Cytochrome P450 11B2, mitochondrial
X2	CHEMBL608140	1,574	0,708	0,865	Cytochrome P450 11B2, mitochondrial
X2	CHEMBL1649605	1,559	0,759	0,8	Cytochrome P450 11B2, mitochondrial
X2	CHEMBL599222	1,551	0,885	0,666	Cytochrome P450 11B2, mitochondrial
X2	CHEMBL1651492	1,545	0,745	0,8	Cytochrome P450 11B2, mitochondrial
X2	CHEMBL1651495	1,531	0,731	0,8	Cytochrome P450 11B2, mitochondrial
X2	CHEMBL599601	1,522	0,832	0,691	Cytochrome P450 11B2, mitochondrial
X2	CHEMBL598390	1,51	0,71	0,8	Cytochrome P450 11B2, mitochondrial
X2	CHEMBL1651493	1,508	0,709	0,8	Cytochrome P450 11B2, mitochondrial
X2	CHEMBL608437	1,507	0,707	0,8	Cytochrome P450 11B2, mitochondrial
X3	CHEMBL681	1,89	0,895	0,996	Cytochrome P450 11B2, mitochondrial
X3	CHEMBL23731	1,89	0,895	0,996	Cytochrome P450 11B2, mitochondrial
X3	CHEMBL611680	1,72	0,887	0,833	Cytochrome P450 11B2, mitochondrial
X3	CHEMBL1649605	1,594	0,795	0,8	Cytochrome P450 11B2, mitochondrial
X3	CHEMBL1651492	1,583	0,784	0,8	Cytochrome P450 11B2, mitochondrial
X3	CHEMBL1651495	1,562	0,763	0,8	Cytochrome P450 11B2, mitochondrial
X3	CHEMBL599601	1,561	0,871	0,691	Cytochrome P450 11B2, mitochondrial
X3	CHEMBL598390	1,545	0,745	0,8	Cytochrome P450 11B2, mitochondrial
X3	CHEMBL1651493	1,544	0,744	0,8	Cytochrome P450 11B2, mitochondrial
X3	CHEMBL608437	1,542	0,742	0,8	Cytochrome P450 11B2, mitochondrial
X3	CHEMBL212741	1,532	0,734	0,798	Cytochrome P450 11B2, mitochondrial
X3	CHEMBL377770	1,532	0,733	0,798	Cytochrome P450 11B2, mitochondrial
X3	CHEMBL1651494	1,53	0,731	0,8	Cytochrome P450 11B2, mitochondrial
X3	CHEMBL1328035	1,52	0,854	0,666	Cytochrome P450 11B2, mitochondrial
X3	CHEMBL162496	1,517	0,851	0,666	Cytochrome P450 11B2, mitochondrial
X3	CHEMBL1428763	1,517	0,851	0,666	Cytochrome P450 11B2, mitochondrial
X3	CHEMBL1444873	1,517	0,851	0,666	Cytochrome P450 11B2, mitochondrial
X3	CHEMBL599222	1,515	0,849	0,666	Cytochrome P450 11B2, mitochondrial
X3	CHEMBL599623	1,505	0,839	0,666	Cytochrome P450 11B2, mitochondrial
X3	CHEMBL599622	1,504	0,838	0,666	Cytochrome P450 11B2, mitochondrial
X3	CHEMBL610501	1,5	0,835	0,666	Cytochrome P450 11B2, mitochondrial
X5	CHEMBL599601	1,668	0,919	0,749	Cytochrome P450 11B2, mitochondrial
X5	CHEMBL681	1,514	0,803	0,711	Cytochrome P450 11B2, mitochondrial
X5	CHEMBL23731	1,514	0,803	0,711	Cytochrome P450 11B2, mitochondrial
X5	CHEMBL611680	1,51	0,903	0,607	Cytochrome P450 11B2, mitochondrial
X6	CHEMBL681	1,893	0,913	0,98	Cytochrome P450 11B2, mitochondrial
X6	CHEMBL23731	1,893	0,913	0,98	Cytochrome P450 11B2, mitochondrial
X6	CHEMBL611680	1,673	0,84	0,833	Cytochrome P450 11B2, mitochondrial
X6	CHEMBL599222	1,554	0,888	0,666	Cytochrome P450 11B2, mitochondrial
X6	CHEMBL599622	1,552	0,885	0,667	Cytochrome P450 11B2, mitochondrial
X6	CHEMBL599623	1,55	0,884	0,667	Cytochrome P450 11B2, mitochondrial
X6	CHEMBL1651492	1,549	0,75	0,8	Cytochrome P450 11B2, mitochondrial
X6	CHEMBL608140	1,529	0,862	0,667	Cytochrome P450 11B2, mitochondrial
X6	CHEMBL610501	1,528	0,864	0,664	Cytochrome P450 11B2, mitochondrial
X6	CHEMBL598390	1,515	0,715	0,8	Cytochrome P450 11B2, mitochondrial
X6	CHEMBL1651493	1,514	0,714	0,8	Cytochrome P450 11B2, mitochondrial
X6	CHEMBL591465	1,513	0,941	0,571	Cytochrome P450 11B2, mitochondrial
X6	CHEMBL608437	1,512	0,712	0,8	Cytochrome P450 11B2, mitochondrial
X6	CHEMBL598606	1,51	0,939	0,571	Cytochrome P450 11B2, mitochondrial
X6	CHEMBL463582	1,507	0,842	0,664	Cytochrome P450 11B2, mitochondrial
X6	CHEMBL1651494	1,503	0,703	0,8	Cytochrome P450 11B2, mitochondrial
X7	CHEMBL681	1,682	0,702	0,981	Cytochrome P450 11B2, mitochondrial

X7	CHEMBL23731	1,682	0,702	0,981	Cytochrome P450 11B2, mitochondrial
X8	CHEMBL681	1,832	0,853	0,978	Cytochrome P450 11B2, mitochondrial
X8	CHEMBL23731	1,832	0,853	0,978	Cytochrome P450 11B2, mitochondrial
X8	CHEMBL599622	1,548	0,881	0,667	Cytochrome P450 11B2, mitochondrial
X8	CHEMBL599623	1,546	0,879	0,667	Cytochrome P450 11B2, mitochondrial
X8	CHEMBL599222	1,544	0,878	0,666	Cytochrome P450 11B2, mitochondrial
X8	CHEMBL608140	1,526	0,859	0,667	Cytochrome P450 11B2, mitochondrial
X10	CHEMBL599601	1,533	0,784	0,75	Cytochrome P450 11B2, mitochondrial
X18	CHEMBL599601	1,6	0,851	0,749	Cytochrome P450 11B2, mitochondrial
X18	CHEMBL681	1,512	0,801	0,711	Cytochrome P450 11B2, mitochondrial
X18	CHEMBL23731	1,512	0,801	0,711	Cytochrome P450 11B2, mitochondrial
X19	CHEMBL599601	1,637	0,887	0,749	Cytochrome P450 11B2, mitochondrial
X19	CHEMBL681	1,541	0,831	0,711	Cytochrome P450 11B2, mitochondrial
X19	CHEMBL23731	1,541	0,831	0,711	Cytochrome P450 11B2, mitochondrial
X20	CHEMBL599601	1,766	0,909	0,857	Cytochrome P450 11B2, mitochondrial
X20	CHEMBL681	1,681	0,851	0,83	Cytochrome P450 11B2, mitochondrial
X20	CHEMBL23731	1,681	0,851	0,83	Cytochrome P450 11B2, mitochondrial
X20	CHEMBL611680	1,585	0,896	0,688	Cytochrome P450 11B2, mitochondrial
X20	CHEMBL610794	1,564	0,857	0,707	Cytochrome P450 11B2, mitochondrial
X20	CHEMBL1651492	1,549	0,882	0,667	Cytochrome P450 11B2, mitochondrial
X20	CHEMBL591465	1,549	0,843	0,707	Cytochrome P450 11B2, mitochondrial
X20	CHEMBL598606	1,546	0,839	0,707	Cytochrome P450 11B2, mitochondrial
X20	CHEMBL598390	1,545	0,879	0,666	Cytochrome P450 11B2, mitochondrial
X20	CHEMBL1651493	1,545	0,878	0,666	Cytochrome P450 11B2, mitochondrial
X20	CHEMBL377770	1,544	0,877	0,666	Cytochrome P450 11B2, mitochondrial
X20	CHEMBL608437	1,544	0,877	0,666	Cytochrome P450 11B2, mitochondrial
X20	CHEMBL212741	1,543	0,877	0,667	Cytochrome P450 11B2, mitochondrial
X20	CHEMBL608140	1,534	0,768	0,767	Cytochrome P450 11B2, mitochondrial
X20	CHEMBL1651494	1,52	0,854	0,667	Cytochrome P450 11B2, mitochondrial
X20	CHEMBL599622	1,52	0,753	0,767	Cytochrome P450 11B2, mitochondrial
X20	CHEMBL599623	1,518	0,752	0,767	Cytochrome P450 11B2, mitochondrial
X20	CHEMBL1649605	1,503	0,837	0,667	Cytochrome P450 11B2, mitochondrial
X21	CHEMBL599601	2	1	1	Cytochrome P450 11B2, mitochondrial
X21	CHEMBL611680	1,782	0,982	0,8	Cytochrome P450 11B2, mitochondrial
X21	CHEMBL162496	1,681	0,967	0,714	Cytochrome P450 11B2, mitochondrial
X21	CHEMBL349822	1,629	0,914	0,714	Cytochrome P450 11B2, mitochondrial
X21	CHEMBL599223	1,593	0,968	0,625	Cytochrome P450 11B2, mitochondrial
X21	CHEMBL9298	1,554	0,931	0,623	Cytochrome P450 11B2, mitochondrial
X21	CHEMBL31215	1,554	0,931	0,623	Cytochrome P450 11B2, mitochondrial
X21	CHEMBL599222	1,552	0,839	0,714	Cytochrome P450 11B2, mitochondrial
X21	CHEMBL598399	1,547	0,868	0,679	Cytochrome P450 11B2, mitochondrial
X21	CHEMBL3099695	1,542	0,918	0,624	Cytochrome P450 11B2, mitochondrial
X21	CHEMBL3099701	1,53	0,907	0,623	Cytochrome P450 11B2, mitochondrial
X21	CHEMBL3099702	1,529	0,907	0,622	Cytochrome P450 11B2, mitochondrial
X21	CHEMBL3099683	1,511	0,888	0,622	Cytochrome P450 11B2, mitochondrial
X21	CHEMBL681	1,502	0,791	0,712	Cytochrome P450 11B2, mitochondrial
X21	CHEMBL23731	1,502	0,791	0,712	Cytochrome P450 11B2, mitochondrial
X23	CHEMBL681	1,74	0,922	0,817	Cytochrome P450 11B2, mitochondrial
X23	CHEMBL23731	1,74	0,922	0,817	Cytochrome P450 11B2, mitochondrial
X24	CHEMBL599601	1,728	0,978	0,75	Cytochrome P450 11B2, mitochondrial
X24	CHEMBL211207	1,621	0,907	0,714	Cytochrome P450 11B2, mitochondrial
X24	CHEMBL611680	1,558	0,955	0,603	Cytochrome P450 11B2, mitochondrial
X24	CHEMBL681	1,542	0,83	0,712	Cytochrome P450 11B2, mitochondrial
X24	CHEMBL23731	1,542	0,83	0,712	Cytochrome P450 11B2, mitochondrial
X3	CHEMBL377770	1,532	0,733	0,798	Aromatase
X3	CHEMBL441367	1,531	0,733	0,798	Aromatase
X3	CHEMBL162496	1,517	0,851	0,666	Aromatase
X3	CHEMBL14192	1,514	0,716	0,798	Aromatase
X3	CHEMBL1203757	1,51	0,844	0,666	Aromatase
X20	CHEMBL14192	1,569	0,902	0,667	Aromatase
X20	CHEMBL377770	1,544	0,877	0,666	Aromatase
X20	CHEMBL441367	1,543	0,876	0,667	Aromatase
X21	CHEMBL162496	1,681	0,967	0,714	Aromatase

X21	CHEMBL9298	1,554	0,931	0,623	Aromatase
X21	CHEMBL31215	1,554	0,931	0,623	Aromatase
X21	CHEMBL468419	1,554	0,931	0,623	Aromatase
X21	CHEMBL481092	1,554	0,931	0,623	Aromatase
X21	CHEMBL1203762	1,533	0,917	0,616	Aromatase
X21	CHEMBL162834	1,526	0,904	0,623	Aromatase
X24	CHEMBL211207	1,621	0,907	0,714	Aromatase
X24	CHEMBL1203766	1,545	0,922	0,623	Aromatase

Table S3: Docking scores of the investigated compounds into the PDB crystal structures 3EQM (CYP19A1), 6M7X (CYP11B1) and 4FDH (CYP11B2). Docking calculations of compounds X11-X17 into the selected conformation of CYP19A1 did not produce any pose, due to the steric hindrance deriving from the structural decorations on the investigated scaffold (see Figure 2 of the main article).

Compound ID	*Docking Score (3QDM, CYP19A1)*	*Docking Score (6M7X, CYP11B1)*	*Docking Score (4FDH, CYP11B2)*
X1	-14,13	-13,11	-13,97
X2	-12,50	-11,98	-12,59
X3	-13,30	-12,15	-13,27
X4	-13,07	-11,13	-12,78
X5	-14,01	-11,31	-13,05
X6	-12,82	-12,24	-13,72
X7	-12,57	-11,95	-11,33
X8	-11,47	-9,75	-12,83
X9	-10,24	-5,62	-2,26
X10	-14,75	-11,01	-12,47
X11	*n.e.*	*n.e.*	*n.e.*
X12	*n.e.*	*n.e.*	*n.e.*
X13	*n.e.*	*n.e.*	*n.e.*
X14	*n.e.*	*n.e.*	*n.e.*
X15	*n.e.*	*n.e.*	*n.e.*
X16	*n.e.*	*n.e.*	*n.e.*
X17	*n.e.*	*n.e.*	*n.e.*
X18	-10,99	-13,09	-11,32
X19	-13,14	-12,67	-11,79
X20	-13,19	-11,66	-12,34
X21	-14,19	-14,14	-13,81
X22	*n.e.*	*n.e.*	*n.e.*
X23	-11,81	-10,80	-12,85
X24	-13,16	-12,84	-12,70
0T3	-12,65		-15,74
JD7	-14,03	-14,99	
ASD	-17,17		

Note: n.e. – not evaluated. Docking pose was not obtained.

Figure S1: Binding mode predicted for (S)- and (R)-fadrozole into the CYP19A1 binding site (PDB ID: 3EQM) (**panels a** and **b**). **Panels c** and **d** report the predicted binding mode of compound **X21** into the CYP11B1 (PDB ID: 6M7X) and CYP11B2 (PDB ID: 4FDH) binding sites. **Panels e** to **g** report redocking results of ASD (panel e), JD7 (panel f) and 0T3 (panel g) into their parent crystallographic complex (*i.e.*, PDB ID: 3EQM, 6M7X and 4FDH, respectively), superimposed to their native conformation.

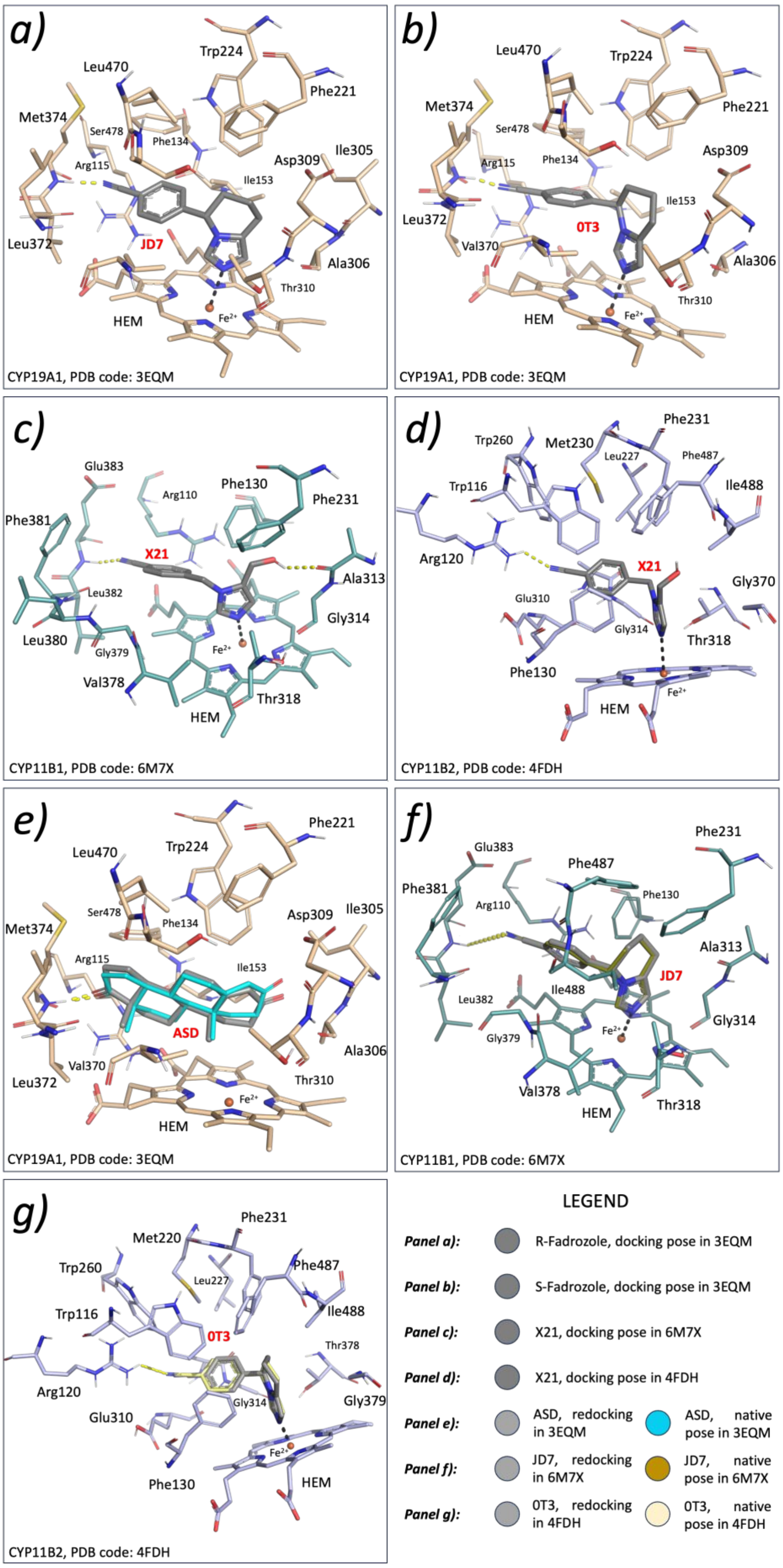

Figure S2: Titration curves of compound **X21** against CYP19A1, CYP1A2 and CYP3A4, with their respective controls.

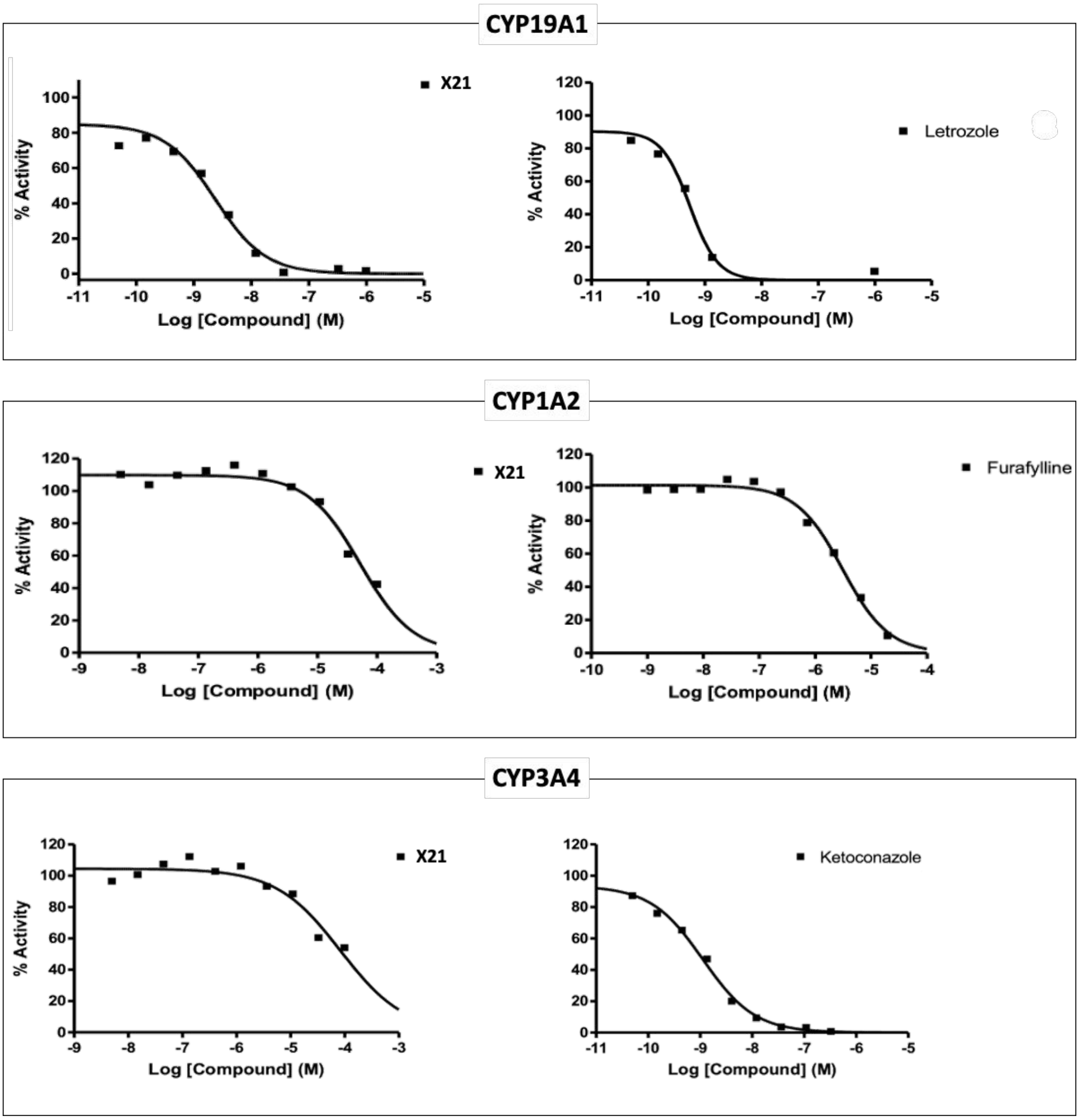

Figure S3: Antiproliferative *in vitro* effects of fadrozole on human MCF-7 (Estrogen Receptor +) at 24h (green line), 48h (blue line) and 72h (red line).

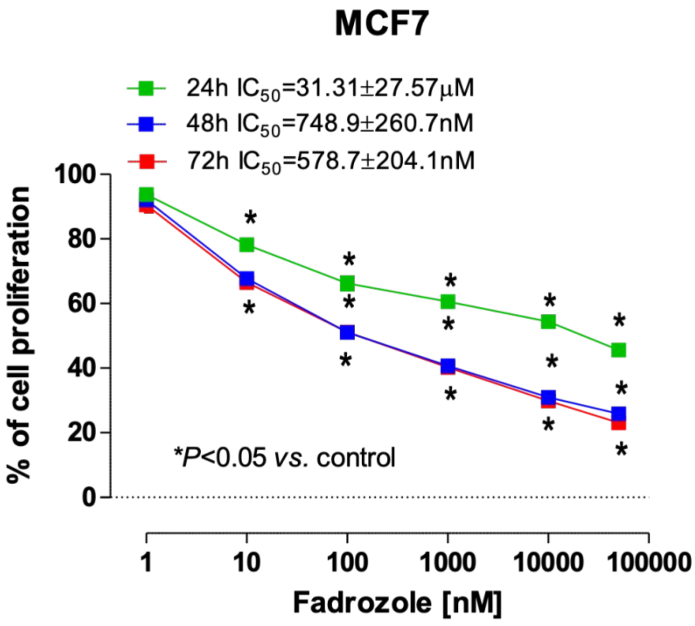

Figure S4: Titration curves of compound **X21** and the reference compound E-4031 and tetrodoxin, against *h*ERG and Nav1.5 (manual patch clamp assays), respectively.

Assays on *h*ERG

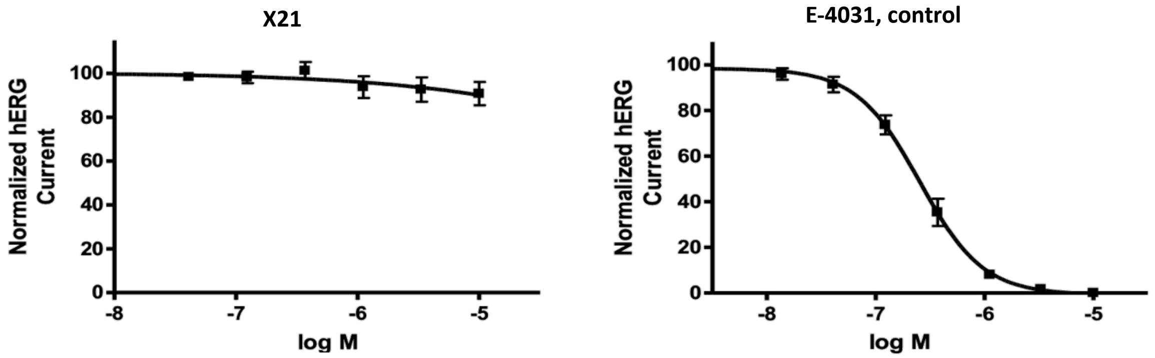

Assays on Nav1.5

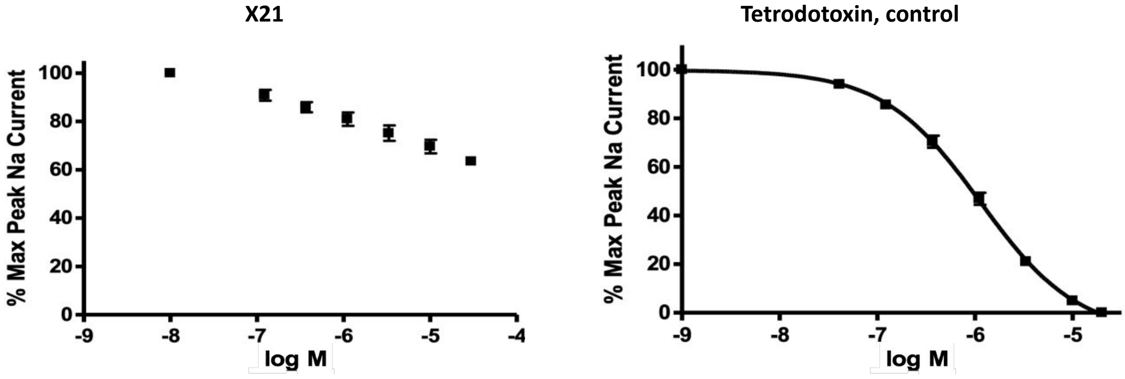

Supplement: Supplementary file 1 — pt3c00183_si_001.pdf [file pt3c00183_si_001.pdf]
